# Cross-talk between disulfidptosis and immune check point genes defines the tumor microenvironment for the prediction of prognosis and immunotherapies in glioblastoma

**DOI:** 10.1038/s41598-024-52128-x

**Published:** 2024-02-16

**Authors:** Yanjun Zhou, Xue Qin, Qunchao Hu, Shaolei Qin, Ran Xu, Ke Gu, Hua Lu

**Affiliations:** 1 Department of Radiotherapy and Oncology, Affiliated Hospital of Jiangnan University, Wuxi, 214000 Jiangsu China; 2 Wuxi School of Medicine, Jiangnan University, Wuxi, 214122 Jiangsu China; 3 Department of Radiation Oncology, Shanghai Tongren Hospital, Shanghai Jiao Tong University School of Medicine, China Shanghai,; 4 Department of Neurosurgery, Affiliated Hospital of Jiangnan University, Wuxi, 214125 Jiangsu China

**Keywords:** CNS cancer, Prognostic markers, Cancer, Computational biology and bioinformatics

## Abstract

Disulfidptosis is a condition where dysregulated NAPDH levels and abnormal accumulation of cystine and other disulfides occur in cells with high SLC7A11 expression under glucose deficiency. This disrupts normal formation of disulfide bonds among cytoskeletal proteins, leading to histone skeleton collapse and triggering cellular apoptosis. However, the correlation between disulfidptosis and immune responses in relation to glioblastoma survival rates and immunotherapy sensitivity remains understudied. Therefore, we utilized The Cancer Genome Atlas and The Chinese Glioma Genome Atlas to identify disulfidptosis-related immune checkpoint genes and established an overall survival (OS) prediction model comprising six genes: CD276, TNFRSF 14, TNFSF14, TNFSF4, CD40, and TNFRSF18, which could also be used for predicting immunotherapy sensitivity. We identified a cohort of glioblastoma patients classified as high-risk, which exhibited an upregulation of angiogenesis, extracellular matrix remodeling, and epithelial-mesenchymal transition as well as an immunosuppressive tumor microenvironment (TME) enriched with tumor associated macrophages, tumor associated neutrophils, CD8^ +^ T-cell exhaustion. Immunohistochemical staining of CD276 in 144 cases further validated its negative correlation with OS in glioma. Disulfidptosis has the potential to induce chronic inflammation and an immunosuppressive TME in glioblastoma.

## Introduction

Glioblastoma, a rapidly progressing grade IV malignant glioma, is the third most common histopathology among central nervous system (CNS) tumors, accounting for 14.2% of all primary brain and CNS tumors^1^. The relative survival rates for glioblastoma patients are significantly low, with only 6.9% of individuals surviving beyond five years after diagnosis. Effective treatments for progressive or recurrent glioblastoma remain a challenge, with only about 8% of patients showing objective responses to PD-1 immune checkpoint inhibitors (CheckMate 143, NCT 02017717). Predicting survival outcome for glioblastoma patients with prognostic genes is crucial to develop a risk classification strategy and enhance therapy precision, which is currently limited.

Cell death is crucial for maintaining internal homeostasis and regulating biological development. Targeted modulation of cell death pathways is a key focus in cancer therapy to effectively eliminate malignant cells^2^. In a recent study by Liu et al., a novel form of cell death called disulfidptosis was identified^3^. Nicotinamide adenine dinucleotide (NADPH) depletion and abnormal accumulation of cystine and disulfides in glucose-deficient, solute carrier family 7 member 11 (SLC7A11) high-expressing cells disrupt the formation of disulfide bonds in cytoskeletal proteins. This destabilizes histone skeletons and triggers cellular apoptosis, a process known as disulfidptosis. However, the role of disulfidptosis in glioblastoma is not well understood.

The interaction between tumor cells and the tumor microenvironment (TME) plays a critical role in tumor progression and treatment response^4^. Cancer cells express inhibitory ligands that suppress T cell function, leading to tumor tolerance and immune evasion^5^. In recent years, immune checkpoint therapies, specifically monoclonal antibodies targeting PD-1 and CTLA-4, have emerged as promising immunotherapeutic approaches for cancer treatment^6^. Building upon the early success of immune checkpoint therapy, targeting additional coinhibitory and costimulatory molecules to activate the antitumor immune response holds great promise as a therapeutic strategy^7^. Understanding and characterizing the TME is crucial for identifying novel therapeutic targets in glioblastoma.

Complex crosstalk between cell death and immune cells is observed^8^. The relationship between disulfidptosis and antitumor immunity has not been explored. Investigating the coexpression of disulfidptosis-related genes and immune checkpoint genes can provide insights into the correlation between disulfidptosis and the immune response.

This research aims to investigate the impact of disulfidptosis-related immune checkpoint genes (DICR genes) on glioblastoma patients. A risk score model based on DRIC genes was constructed to assess the prognostic value and immunotherapy sensitivity in glioblastoma. The study aims to uncover molecular mechanisms underlying the role of DRIC genes in glioblastoma and propose a new prediction model for glioblastoma immune therapy.

## Results

### Construction and validation of disulfidptosis related immune checkpoint prognostic signature

**Figure 1:**
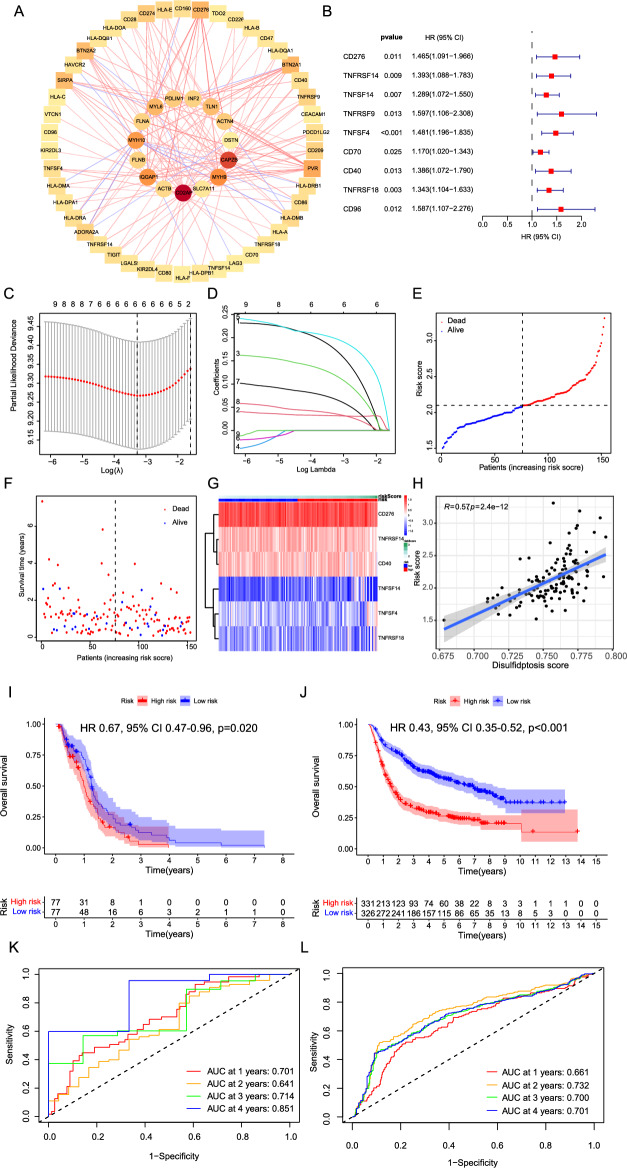
Construction and validation of disulfidptosis related immune checkpoint prognostic signature. (**A**) Correlation analysis demonstrates the significant association of immune checkpoint genes with genes involved in disulfidptosis, with an absolute correlation coefficient exceeding 0.3 and a *p*-value below 0.05 in the TCGA-glioblastoma dataset. (**B**) Forest plot representing the significant association with overall survival of 9 immune checkpoint genes identified through univariate Cox regression analysis in the TCGA-glioblastoma dataset. (**C**) This figure shows the relationship between the partial likelihood deviance and different levels of regularization for the Lasso model. The y-axis represents the partial likelihood deviance, which measures the goodness-of-fit of the Lasso model. A lower value indicates a better fit. The x-axis corresponds to the log λ, which controls the amount of regularization applied in the Lasso analysis. Higher values of log λ result in greater regularization. The dashed vertical line refers to the optimal λ value. (**D**) The coefficients obtained from Lasso-Cox analysis. The y-axis represents the coefficients, while the x-axis represents the log λ. Genes with non-zero coefficients are used to establish of prognostic prediction model. (**E**) The scatter plot shows the relationship between the patients (x-axis) and their risk score (y-axis). Red points indicate deceased patients, while blue points represent surviving patients. The dashed line represents the division of the TCGA-glioblastoma cohort into high- and low- risk groups based on the median value of the risk score (**F**) The scatter plot with the x-axis representing the patients with increasing risk score and the y-axis representing survival time. Red points indicate cases of death, while blue points represent cases of survival. (**G**) The heat map illustrates the expression patterns of six modeling genes based on their risk scores. (**H**) The correlation between risk score and disulfidptosis score calculated using single sample gene set enrichment analysis. Kaplan–Meier survival curves illustrating the overall survival of high- and low-risk groups in both the TCGA-glioblastoma cohort (**I**) and CGGA cohort (**J**). The ROC curves for years 1, 2, 3, and 4 in the TCGA-glioblastoma cohort (**K**) and CGGA cohort (**L**) were displayed. HR, Hazard Ratio; CI, Confidence Interval; CGGA, Chinese Glioma Genome Atlas; TCGA, The Cancer Genome Atlas; ROC, Receiver Operating Characteristic.

The DRIC genes were identified with an absolute correlation coefficient > 0.3 and a p-value < 0.05 (Fig. 1A and Supplementary Table 1). The results of univariate Cox analysis showed that increased expression of CD276 (HR 1.47, 95% confidence interval (CI) 1.10–1.97, *p* = 0.011), TNFRSF14 (HR 1.39, 95% CI 1.09–1.78, *p* = 0.009), TNFSF14 (HR 1.29, 95% CI 1.07–1.55, *p* = 0.007), TNFRSF9 (HR 1.60, 95% CI 1.11–2.31, *p* = 0.013), TNFSF4 (HR 1.48, 95% CI 1.20–1.84, *p* < 0.001), CD70 (HR 1.17, 95% CI 1.02–1.34, *p* = 0.025), CD40 (HR 1.39, 95% CI 1.07–1.79, *p* = 0.013), TNFRSF18 (HR 1.34, 95% CI 1.10–1.63, *p* = 0.003), and CD96 (HR 1.59, 95% CI 1.11–2.28, *p* = 0.012) were associated with poor prognoses of glioblastoma (Fig. 1B). To address multicollinearity among DRIC genes derived from the univariate Cox and simplify the prognostic model, we performed LASSO-Cox analysis. Figure 1C displays the optimal tuning parameter λ, which was determined to be 0.038, resulting in the minimum partial likelihood deviance. The Lasso-Cox analysis identified six DRIC genes with non-zero coefficients, which constitute the prognostic model (Fig. 1D). The expression values and coefficients of the DRIC genes in the prognostic model can be found in Supplementary Table 2. The risk score for each patient was calculated using the formula: Risk Score = CD276 × 0.175 + TNFRSF14 × 0.032 + TNFSF14 × 0.120 + TNFSF4 × 0.199 + CD40 × 0.068 + TNFRSF18 × 0.036. The TCGA glioblastoma cohort was then divided into low-risk and high-risk groups based on the median risk score (Fig. 1E). A positive correlation was observed between the risk score and the occurrence of death events, indicating an increase in death events with higher risk scores (Fig. 1F). The expression of the 6 DRIC genes according to the risk score is depicted in Fig. 1G. The disulfidptosis score was calculated using ssGSEA, and a strong relationship was observed between the risk score and the disulfidptosis score (R = 0.57, *p* < 0.001, Fig. 1H). Moreover, the risk score exhibited a significant increase in patients older than 60 years compared to patients aged 60 years or younger (*p* = 0.044, Supplementary Fig. 1A). Notably, the risk score showed a significant increase in IDH wild-type (IDH WT) compared to IDH mutant patients (*p* < 0.001, Supplementary Fig. 1C). The risk score did not exhibit any differences across different ethnicities and genders (Supplementary Fig. 1B, D). Furthermore, patients classified in the high-risk group exhibited a significantly poorer OS than those assigned to the low-risk group in the TCGA glioblastoma cohort (HR 0.67, 95% CI 0.47–0.96, *p* = 0.02, Fig. 1I) and the Chinese Glioma Genome Atlas (CGGA) cohort (HR 0.43, 95% CI 0.35–0.52, *p* < 0.001, Fig. 1J). In addition, both primary glioblastoma and recurrent glioblastoma subgroups exhibit significantly worse OS in the high-risk group compared to the low-risk group in the CGGA cohort (Supplementary Fig. 2A). Moreover, the treatment to survival outcome in the high-risk group was significantly worse compared to the low-risk group in GSE13041 (Supplementary Fig. 2C). To assess the predictive performance of the model, Receiver Operating Characteristic (ROC) curves were generated. In the TCGA-glioblastoma cohort, the AUC values were 0.701 at 1 year, 0.641 at 2 years, 0.714 at 3 years, and 0.851 at 4 years (Fig. 1K). Similarly, in the testing CGGA cohort, the model demonstrated AUC values of 0.661 at 1 year, 0.732 at 2 years, 0.700 at 3 years, 0.701 at 4 years (Fig. 1L). The AUC was about 0.6 in the CGGA-glioblastoma cohort (Supplementary Fig. 2B). The AUC was 0.702 in the GSE13041 cohort (Supplementary Fig. 2D). Moreover, to compare the OS between the high- and low-expression groups, gene expression levels of CD40, CD276, TNFRSF14, TNFRSF18, TNFSF4, and TNFSF14 were used for classification (Supplementary Fig. 3A–F). In the TCGA cohort, high expression of CD40, CD276, TNFRSF14, TNFRSF18, TNFSF4, and TNFSF14 was associated with a worse prognosis. Similar associations were observed in the CGGA-glioblastoma cohort for CD40, CD276, TNFRSF14, and TNFSF14, except for TNFSF4 and TNFRSF18 (Supplementary Fig. 4). These findings highlight the robust nature and applicability of the model in predicting the OS of glioblastoma patients across various time intervals.

### Construction and validation of a nomogram

**Figure 2:**
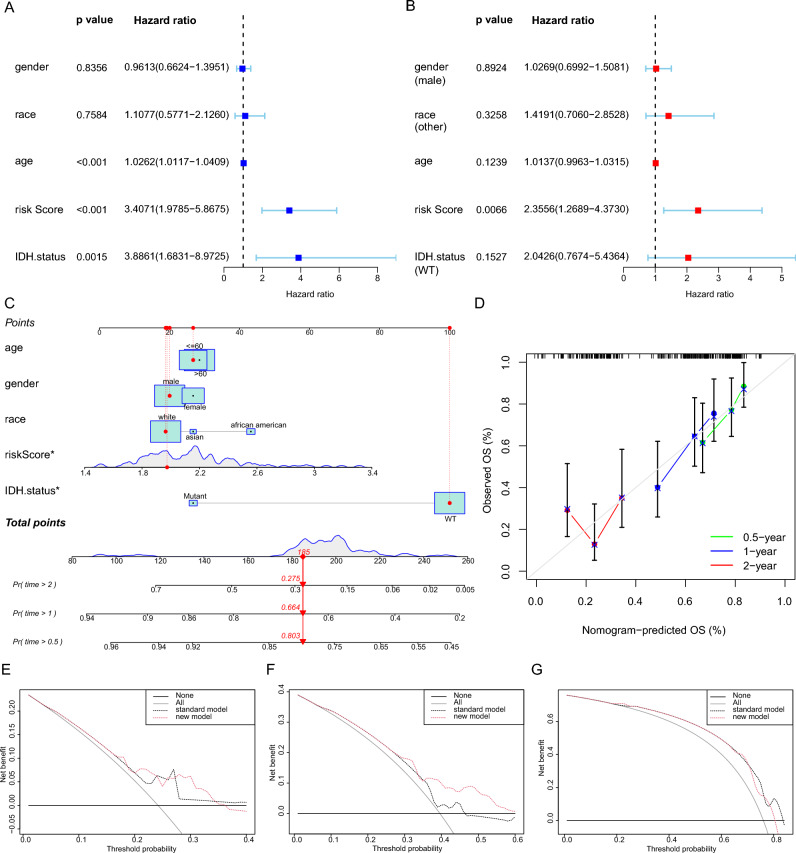
Development and validation of a nomogram. (**A**) Univariate Cox regression analysis of the impact of clinical and pathological factors, as well as risk score, on overall survival in the TCGA-glioblastoma cohort. (**B**) Multivariate Cox regression analysis of the impact of clinical and pathological factors, as well as risk score, on overall survival in the TCGA-glioblastoma cohort. (**C**) Nomogram for predicting overall survival at 0.5, 1, and 2 years based on the age, gender, race, risk score and IDH status in the TCGA-glioblastoma cohort. (**D**) The calibration curves of the nomogram model for predicting overall survival at 0.5, 1, and 2 years. The x-axis represents the predicted probability of survival, while the y-axis shows the actual observed probability of survival. The Decision Curve Analysis shows the net benefit of each model at different time points: 0.5 years (**E**), 1 year (**F**), and 2 years (**G**). The new model represents a nomogram that includes a risk score, while the standard model represents a model without a risk score.

To confirm the independent relationship between the risk score and OS, univariate and multivariate Cox regression analyses were performed. The risk score and IDH status (WT) were independently associated with OS in the univariate Cox analysis (HR: 3.40, 95% CI: 1.98–5.87, *p* < 0.001; HR: 3.89, 95% CI: 1.68–8.97, *p* = 0.002, respectively, Fig. 2A). Moreover, in the multivariate Cox regression analysis, the risk score exhibited a significant association with OS (HR: 2.36, 95% CI: 1.27–4.37, *p* = 0.007, Fig. 2B). Collectively, our findings suggest that the risk score holds comparable prognostic value to conventional clinicopathological variables, such as IDH status, and can independently predict OS in glioblastoma patients. Based on these results, we developed a feasible nomogram (Fig. 2C). For instance, a male glioblastoma patient of white ethnicity with WT IDH status, aged above 60 years, and a risk score of 2.0 would accumulate a total of 185 points. Using the nomogram, this patient's estimated probabilities of OS were approximately 0.803 at 0.5 years, 0.664 at 1 year, and 0.275 at 2 years (Fig. 2C). A calibration plot was generated to assess the coherence prediction of the nomogram model and the observed outcomes (Fig. 2D). Moreover, the practicality of the nomogram in a clinical setting was assessed by DCA plots (Fig. 2E–G). The nomogram exhibited superior net benefit in predicting 1-year OS compared to the conventional model (Fig. 2F).

### Identification of differential expression genes and functional enrichment analysis

**Figure 3:**
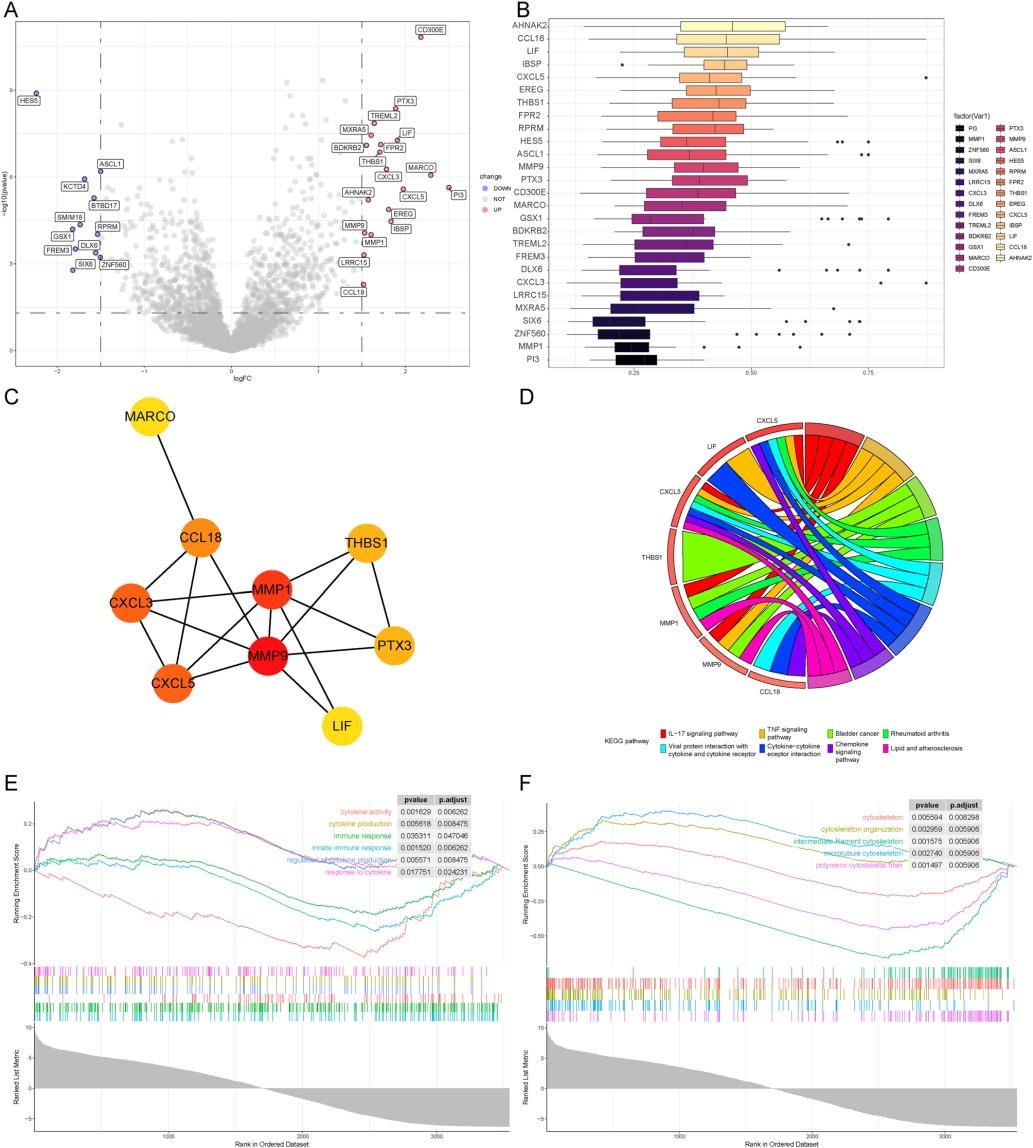
Identification of differential expression genes and functional enrichment analysis. (**A**) Volcano plot illustrating Differentially Expressed Genes (DEGs) using a significance threshold of adjusted *p* value < 0.05 and an absolute log2 fold change exceeding 1.5. The hub genes among the DEGs identified by "GOSemSim" analysis (**B**) and "closeness" in Cytoscape CytoHubba (**C**). (**D**) Kyoto Encyclopedia of Genes and Genomes (KEGG) enrichment analysis based on DEGs in the TCGA-glioblastoma cohort. (**E**, **F**) Gene set enrichment analysis (GSEA) analysis in the TCGA-glioblastoma cohort.

To explore the mechanisms underlying the poorer prognosis in the high-risk group, DEGs and functional enrichment were performed (Fig. 3A). A total of 11 genes were downregulated and 19 genes showed elevated expression levels in the high-risk group (Fig. 3A and Supplementary Table 3). Hub genes were identified among DEGs by “GOSemSim” analysis (Fig. 3B) and by the algorithm “closeness” in Cytoscape CytoHubba in the PPI network (Fig. 3C). The genes MARCO, MMP1, MMP9, CCL18, CXCL3, CXCL5, THBS1, PTX3, and Leukemia inhibitory factor (LIF) have been identified as Hub genes using two different computational methods. Notably, both CXCL3 and CXCL5 were identified as hub genes, indicating the significant involvement of the CXCR2 signaling pathway in the progression of glioblastoma. CXCL3 was found to be a critical cytokine necessary for the growth and proliferation of CD44^ +^ CD24^-^ breast cancer cells with stem cell-like properties^9^. CXCL5 is upregulated in different tumor types and plays a role in promoting angiogenesis, lymphangiogenesis, attracting neutrophils and myeloid-derived suppressor cells (MDSCs), and facilitating primary tumor growth^10^. CXCR2, the receptor for CXCL3 and CXCL5, has been associated with promoting cellular processes such as tumor cell proliferation, migration, invasion, angiogenesis, lymphangiogenesis, and cellular senescence^11^. N2-type neutrophils, which exhibit protumoral characteristics, demonstrate higher expression of CXCR2 than N1-type neutrophils^12^. Moreover, CCL18 production in glioblastoma is primarily attributed to tumor-associated macrophages (TAMs) and cancer-associated fibroblasts (CAFs), with a remarkably elevated level observed in glioblastoma compared to healthy brain tissue, exceeding it by more than 100-fold^13,14^. CCL18 serves as a marker of the M2 macrophage phenotype, which is associated with suppressive TME and tumor immune evasion^14^. Additionally, CCL18 is involved in the recruitment and differentiation of Treg cells^14^. CCL18 can induce proliferation, invasion, and epithelial-to-mesenchymal transition (EMT) in various tumor cells^14^. MMP9 is capable of degrading structural proteins and remodeling the ECM, enabling cancer cells to breach the basement membrane barrier and invade adjacent tissues^15^. Moreover, MMP9 participates in cell proliferation, angiogenesis, and immune inflammation by interacting with diverse substrates^15^. THBS1 encodes thrombospondin-1, a secreted protein in the tumor microenvironment, which is upregulated in response to lactate, leading to increased secretion of TGF-β2 and enhanced migration of glioma cells^16^. Furthermore, the enriched KEGG pathways included the IL-17 signaling pathway, TNF signaling pathway, cytokine-cytokine receptor interaction, chemokine signaling pathway, and other related pathways (Fig. 3D). In addition to the enrichment of cytokine activity and immune response (Fig. 3E), the biofunctions related to the cytoskeleton and filament in the cells (Fig. 3F) were also enriched in the GO enrichment, which might correlate with disulfidptosis.

### Evaluation of the tumor microenvironment

**Figure 4:**
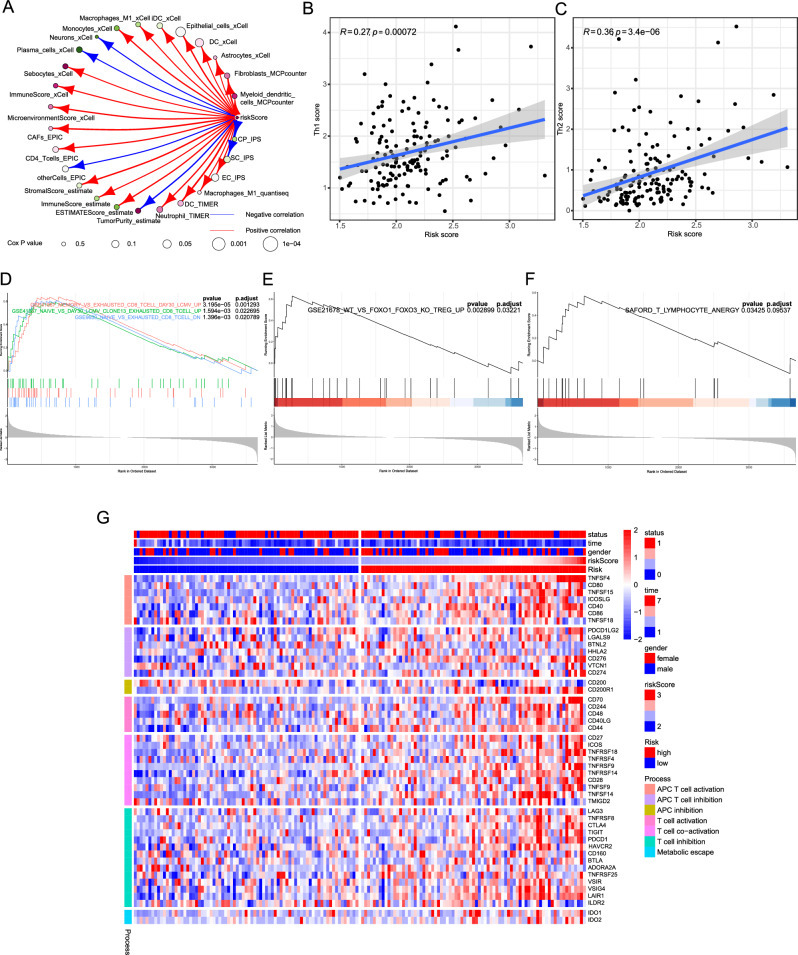
Evaluation of the tumor microenvironment and the adaptive immune response. (**A**) The circle plot illustrates the correlations between risk scores and cellular components as well as scoring in the tumor microenvironment (TME) using different algorithms. The blue line represents a negative correlation, while the red line represents a positive correlation. The circle plot also demonstrates the correlations between cellular components of the TME assessed by various algorithms and overall survival (OS). The size of the circle represents the *p*-value indicating the correlation between cellular components or scores and OS in Cox analysis. The correlation between risk score and Th1 score (**B**), as well as Th2 score (**C**). Evaluation of the functionality of CD8^ +^ T cells (**D**), Treg cells (**E**), and anergy CD4^ +^ (**F**) based on differentially expressed genes using Gene Set Enrichment Analysis through the R Package clusterProfiler version 4.2.2. The analysis incorporated the reference gene set obtained from MSigDB 7.0 (https://www.gsea-msigdb.org/gsea/downloads.jsp). (**G**) The heat map illustrates the evaluation of different immunological processes through the expression of key genes.

The TME's cellular composition was estimated using eight algorithms from the IOBR package. The impact of infiltrating cells and risk score on survival, as determined by Cox regression analysis, is summarized in Supplementary Table 3. Except for the score of CD8^ +^ naive T cells by xCell and SC_IPS, the score of Epithelial cells by xCell, keratinocytes by xCell, MSCs by xCell, CD8^ +^ Tem cells by xCell, dendritic cells (DCs) by xCell, B cells by EPIC, resting DCs by CIBERSORT, EC_IPS, T cells by MCPcounter, class-switched memory B cells by xCell, CD4^ +^ naive T cells by xCell, CD8^+^ T cells by TIMER, and macrophages by TIMER were negatively associated with the risk score and OS (Fig. 4A and Supplementary Table 4).

### Evaluation of the adaptive immune response

Based on the previous research^17,18^, we assessed the correlation between Th1 score and Th2 score in the tumor microenvironment with our risk score. We observed a positive correlation between Th1 score and risk score (R = 0.27, *p* < 0.001, Fig. 4B), as well as a strong positive correlation between Th2 score and risk score (R = 0.36, *p* < 0.001, Fig. 4C). Comparison of the expression profiles of Th1 and Th2 marker genes between high and low-risk groups is depicted in the Supplementary Fig. 5. The association between low Th2 score and favorable prognosis as well as decreased activity of the PD-L1/PD-1 axis indirectly confirms the predictive ability of our risk score for prognosis^17^. Notably, the high-risk group demonstrated an enrichment of CD8^ +^ T cells displaying an exhausted phenotype (Fig. 4D). Furthermore, Treg cell enrichment was identified in the high-risk group (adjusted *p* = 0.03, Fig. 4E). Moreover, GSEA demonstrated that CD4^ +^ T cells did not reach an anergic signature in the high-risk group (adjusted *p* = 0.09, Fig. 4F). The defective T cells may indicate that long-term antigen exposure leads to persistent T-cell activation and upregulation of inhibitory signals. Therefore, based on the expression levels of key genes, we inferred the changes in immunological processes between the high-risk and low-risk groups. As depicted in Fig. 4G, several typical activators, costimulatory factors, and inhibitors were found to be upregulated in the high-risk group, indicating the occurrence of intricate immunological processes.

### Evaluation of the innate immune response and extracellular matrix remodelling

**Figure 5:**
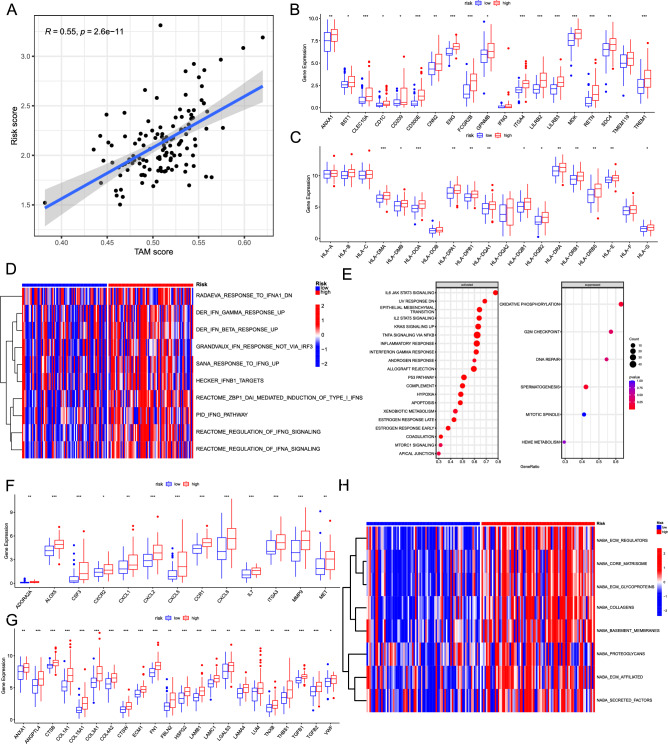
Evaluation of the innate immune response and extracellular matrix remodelling. (**A**) A scatter plot illustrates the correlation between the risk score and TAM score calculated using ssGSEA. Comparison of the expression profiles of monocyte-derived macrophage markers (**B**) and HLA molecules (**C**) in high-risk and low-risk groups of glioblastoma. (**D**) The heatmap represents the comparison of the levels of the IFN signaling pathway between the high-risk and low-risk groups using GSVA. (**E**) GSEA results using the Hallmarker gene set collection. Comparison of the expression profiles of neutrophil markers (**F**) and genes related to extracellular matrix remodeling (**G**) in high-risk and low-risk groups of glioblastoma. (**H**) The heatmap represents the comparison of the levels of the extracellular matrix remodeling between the high-risk and low-risk groups using GSVA. TAM, tumor-associated macrophage; ssGSEA, single-sample gene set enrichment analysis; GSEA, gene set enrichment analysis; GSVA, gene set variation analysis; *, 0.01 < *p* < 0.05; **, 0.001 < *p* < 0.01; ***p < 0.001.

Therefore, we performed a comprehensive analysis of innate immune cell-specific marker expression patterns in the high-risk and low-risk groups within the TME. First, TAMs represent the major cellular component within the glioblastoma TME. To assess TAM abundance, TAM scores were calculated for each sample using ssGSEA, considering a set of 36 upregulated genes associated with TAMs^19^. A notable positive correlation was found between the TAM scores and the risk scores (R = 0.55, *p* < 0.001, Fig. 5A, Supplementary Fig. 6)^19^. Since TAMs are derived from both microglia (MG) and macrophage-derived cells (MDMs) in glioblastoma, we conducted a comprehensive evaluation of marker gene expression in MG and MDMs from high and low-risk groups. In the high-risk group, there was no significant upregulation observed in TMEM119, an established marker of MG (Fig. 5B). Meanwhile, activation markers for MDMs, including FCGR2B and CLEC10A, showed increased expression in the high-risk group (Fig. 5B). Furthermore, genes associated with phagocytic and antigen presentation capabilities in MDMs, such as CD1C and CD209, exhibited enhanced expression in the high-risk group (Fig. 5B). Furthermore, the TAM polarization-related factors were also assessed. Significantly, GPNMB and ANXA1, known to promote TAM polarization and suppress T cell activation^20^, exhibited heightened expression in the high-risk group (Fig. 5B). MDK, its receptor SDC4, and ITGA4 were elevated in the high-risk group (Fig. 5B), enhancing the polarization of TAMs toward an M2-like phenotype^20^. Moreover, genes associated with promoting monocyte migration, survival, and phagocytic activity were also depicted in Fig. 5B. The receptors CD300E and BST1, high in the high-risk group, promote the migration and survival of monocytes^21,22^ (Fig. 5B). The actin-associated regulatory protein CNN2 upregulated in the high-risk group, negatively regulates the motility and phagocytic activity of macrophages^23^. (Fig. 5B). Moreover, the high-risk group exhibited upregulation of LILRB2 and LILRB3, which are known to suppress myeloid cell activation^24^ (Fig. 5B). In addition, the inflammatory genes were also evaluated in the high and low-risk groups. RETN, which was increased in the high-risk group, mediates inflammatory responses^25^ and is upregulated in brain metastasis. The high-risk group showed increased expression of the TREM1 receptor, which plays a crucial role in modulating proinflammatory responses during neuroinflammation in both MGs and MDMs (Fig. 5B)^26,27^.

Notably, several MHC II molecules were elevated in the high-risk group (Fig. 5C). Consistent with these findings, the gene sets linked to antigen processing and presentation were also enriched in the high-risk group (Supplementary Fig. 7). Persistent activation of the IFN signaling pathway, which has been associated with immune suppression and resistance to immune checkpoint blockade, was also observed to be enriched in the high-risk group (Fig. 5D)^28,29^. The high-risk group showed enrichment of the proinflammatory cytokine Interleukin 6 (IL-6) signaling pathway (Fig. 5E), which may potentially be associated with impairing T-cell functionality, promoting immune suppression, and conferring resistance to immune checkpoint blockade therapy^30^. Furthermore, the high-risk group exhibited the activation of EMT^31^ and IL-2 STAT2 signaling, while oxidative phosphorylation, G2M checkpoint, and DNA repair processes were suppressed (Fig. 5E).

Moreover, we identified several neutrophil-related genes enhanced in the high-risk group (Fig. 5F), which may indicate a potent immunosuppressive TME^32^. The elevated expression of CXCL8, an important chemotactic factor for neutrophils, and the increased expression of ITGA3 were found in the high-risk group (Fig. 5F), which facilitates the recruitment of neutrophils to tissues during sepsis^33^. Furthermore, we noted the increased expression of the adenosine receptor ADORA2A in the high-risk group (Fig. 5F), known to suppress the proinflammatory phenotype of neutrophils^34^. ALOX5 was also upregulated in the high-risk group (Fig. 5F), which can enhance the production of LBT4 in neutrophils, promoting tumour cell metastasis^35^. The heightened expression of ARG1 in the high-risk group (Fig. 5F) orchestrated the neutrophil-mediated suppression of antitumor immune responses^36^. Additionally, the differentially expressed cytokines are presented in the Supplementary Fig. 8.

Interestingly, ECM and ECM-associated genes are significantly elevated in the high-risk group, remodeling the ECM, regulating angiogenesis, and influencing tumor immunity^37^ (Fig. 5G). LUM exhibits both pro-metastatic and anti-metastatic properties^38^ (Fig. 5G). CTSB and CTSW are highly expressed in the high-risk group and participate in invasion and metastasis in tumor progression (Fig. 5G)^39^. Additionally, GSVA revealed enrichment of EMC remodeling-related gene sets in the high-risk group, including “NABA COLLAGENS”, “NABA ECM GLYCOPROTEINS”, “NABA ECM REGULATORS”, and so on (Fig. 5H).

### Prediction of sensitivity of immunotherapy

**Figure 6:**
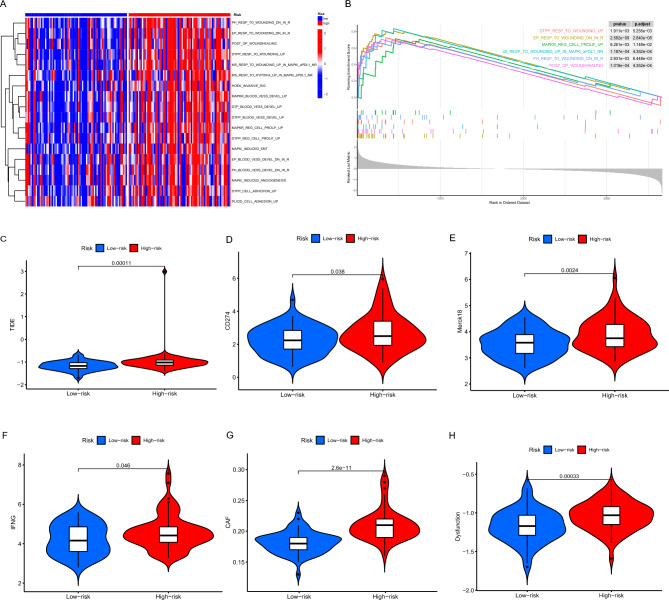
Prediction of sensitivity to immunotherapy. (**A**) GSVA analysis of innate resistance to anti-PD1 therapy gene set collection in high- and low-risk Groups. (**B**) GSEA analysis of innate resistance to anti-PD1 therapy gene set collection in high- and low-risk Groups. The violin plot depicts the comparison of TIDE score (**C**), CD274 (**D**), Merck 18 (**E**), IFNG (**F**), CAFs (**G**), and T-cell dysfunction (**H**) in both the high- and low-risk groups. GSEA, gene set enrichment analysis; GSVA, gene set variation analysis; TIDE, tumor immune dysfunction and exclusion.

Based on the aforementioned findings, which include the dysfunctional states of T cells, suppressive TAMs, suppressive neutrophils, and remodeled EMC within the TME of the high-risk group, these factors represent significant impediments to cancer treatment. Therefore, we utilized our scoring system to predict the efficacy of immunotherapy. Firstly, the high-risk group was significantly associated with innate anti-PD1 resistance^40^ (Fig. 6A,B). Furthermore, the high-risk group exhibited an elevated TIDE (Tumor Immune Dysfunction and Exclusion) score, suggesting the presence of an immune escape phenotype and potential resistance to cancer immunotherapies (Fig. 6C). In the high-risk group, in addition to a higher TIDE score, there was enhanced expression/score of CD274, Merck 18, IFNG, CAFs, and T-cell dysfunction (Fig. 6D–H). Overall, our model's effectiveness in predicting immunotherapy sensitivity was confirmed through two independent evaluation methods.

### Validation the inverse association between survival and CD276 expression in glioma patients

**Figure 7:**
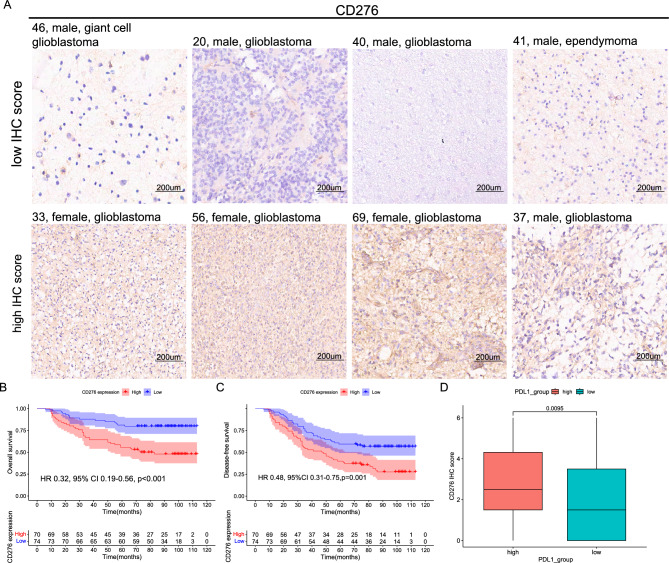
Validation the inverse association between survival and CD276 expression in glioma patients. (**A**) The representative images depicting high expression of CD276 (upper panel) and low expression of CD276 (lower panel) by IHC. Kaplan–Meier analysis for OS (**B**) and DFS (**C**) based on CD276 expression status. (**D**) The boxplot illustrates the comparison of CD276 expression levels in two groups classified based on the median PDL1 expression. IHC, immunohistochemistry; OS, Overall Survival; DFS, Disease-Free Survival; HR, Hazard Ratio; CI, Confidence Interval.

We employed 144 cases of glioma patients from 2008 to 2010 and the expression of CD276 was assessed using immunohistochemistry (IHC). The mean follow-up duration was 72 months. Figure 7A illustrates low expression of CD276 (upper panel) and high expression of CD276 (lower panel). We observed elevated expression of CD276 in patients with shorter OS (HR 0.32, 95% CI 0.19–0.56, *p* < 0.001) and DFS (HR 0.48, 95% CI 0.31–0.75, *p* = 0.001) (Fig. 7B,C). Moreover, the expression of CD276 exhibited a positive correlation with the expression of PD-L1, indicating a potential association between these two molecules (*p* = 0.009, Fig. 7D). In addition, we obtained representative IHC staining images of CD276, TNFSF4, TNFRSF14, CD40, TNFSF14, and TNFRSF18 in both normal tissues and glioblastoma samples from the Human Proteome Atlas (Supplementary Fig. 9).

## Discussion

Our results demonstrate an intense crosstalk between disulfidptosis and immune responses. We identified significant correlations between genes involved in disulfidptosis and immune checkpoint genes. Based on these findings, we established a risk signature comprised of CD276, TNFRSF14, TNFSF14, TNFSF4, CD40, and TNFRSF18 to predict OS and response to immune checkpoint blockade in glioblastoma patients. Validation in the CGGA cohort confirmed the impact of DRIC genes. Additionally, high expression levels of CD276, TNFRSF14, TNFSF14, TNFSF4, and TNFRSF18 have been previously reported to be associated with poor prognosis in glioblastoma^41^. Our risk signature showed an association with the release of inflammatory cytokines, including IL6, IL2, IFN-γ, CCL18, CXCL3, and CXCL5, which may be involved in immune suppression. Therefore, we further evaluated the immune cell infiltration. Our findings revealed that the risk signature was significantly associated with Th2 score, TAM score, enrichment of exhausted CD8 T cells, and enrichment of Treg cells. Additionally, our findings suggest that the high-risk group in glioblastoma exhibits an upregulation of angiogenesis, ECM remodeling and EMT^31^.

While elevated PD-L1 expression is often associated with an antitumor immune response, it does not consistently predict immunotherapy response. Therefore, there is a pressing need to explore biomarkers for predicting treatment response.

Our model genes are also associated with immune therapy response in glioblastoma, including T cell co-stimulatory molecules like TNFRSF18 and TNFSF4. TNFRSF18, also known as GITR, is constitutively expressed at basal levels on naïve or quiescent CD4^ +^ and CD8^ +^ T cells. Upon T-cell activation, GITR expression is upregulated. Notably, Treg cells demonstrate elevated levels of GITR expression^42^. The GITR receptor predominantly serves as a costimulatory molecule for T cells, facilitating their proliferation and augmenting the functionality of effector T cells. However, it has been reported that GITR/GITRL signaling can inhibit the antitumor activity of NK cells^43^. Similar to our findings, Nielsen et al. reported that high expression of GITR on breast cancer cells was associated with lower recurrence-free survival rates^44^. Increased expression of GITR on malignant pleural mesothelioma cells was observed after radiotherapy, and patients with high GITR expression had poorer OS in nonepithelioid malignant pleural mesothelioma^45^. During the chronic stage of LCMV infection, GITR expression is sustained above baseline levels^46^, which further confirms the presence of chronic antigen exposure in the high-risk glioblastoma TME.

TNFSF4 (OX40L), a crucial costimulatory signal for T-cell activation and survival, is expressed by activated APCs, endothelial cells, and DCs upon various APC maturation factors^47^. After antigen recognition, OX40, the receptor for OX40L, is induced and upregulated on T cells. However, upon antigen clearance, the expression of OX40 is downregulated^42^. The OX40-OX40L signaling pathway acts as a crucial costimulatory signal, promoting the proliferation and differentiation of tumor antigen-specific T cells. This pathway contributes to the generation of effector and memory T cell populations. In vitro studies have shown that OX40L/OX40 signaling can make CD4^ +^ T cells resistant to suppression by Tregs^48^ and block Foxp3^ +^ or IL-10^ +^ Treg induction^49^. Recent studies have revealed the diverse functional role of OX40/OX40L signaling in tumor contexts. In breast cancer, the expression of OX40 has been associated with antiapoptotic and tumor-promoting factors, as well as an immune-inhibitory phenotype^50^. While OX40 is not a natural survival factor for Tregs, signaling through OX40 can promote the proliferation or survival of Tregs^51^. These mechanisms may indicate an association between OX40 and an unfavorable prognosis in glioblastoma patients.

In our model, the genes TNFRSF14 and TNFSF14 are also associated with a poorer prognosis in glioblastoma through complex interaction networks. TNFRSF14, also known as HVEM (Herpesvirus Entry Mediator), has a dual functional role as both a receptor and a ligand. As a receptor, HVEM interacts with canonical TNF-related ligands like LIGHT and lymphotoxin-α. Additionally, HVEM acts as a ligand for BTLA (B and T lymphocyte attenuator) and CD160, two immunoglobulin superfamily proteins^52,53^. HVEM, being part of a complex immune regulatory network, can have dual roles in promoting and suppressing immune responses. It is highly expressed on naïve or resting CD4^ +^ and CD8^ +^ T cells, as well as Tregs. LIGHT-HVEM signaling activates the NF-kB transcription program, providing T cells with survival signals and promoting proliferative responses^54^. Furthermore, LIGHT-HVEM signaling enhances IFNγ production in NK cells^55^. However, the induction of tumor-specific T cells by LIGHT alone is insufficient to achieve complete tumor regression^56^. On the other hand, HVEM exhibits immunosuppressive effects. Following binding to HVEM, BTLA undergoes tyrosine phosphorylation and interacts with the TCR complex, as well as phosphatases SHP-1 and SHP-2. This interaction is proposed to attenuate downstream signaling pathways of TCR and dampen T-cell responses^57^. The interaction between BTLA and HVEM plays a negative regulatory role in the homeostatic expansion of memory CD4^ +^ and CD8^ +^ T cells^58^, and inhibits B-cell responses in vivo^59^. Furthermore, CD160 inhibits the activity of immune cells by binding to HVEM^60^. Moreover, the interaction between BTLA and HVEM has been documented to contribute to T-cell survival^61^.

Another molecule in our risk signature is TNFSF14, also known as LIGHT. LIGHT is expressed on activated lymphoid cells, including subsets of APCs and T cells, in both its membrane-bound and soluble forms^42^. In addition to its role in T cell activation, the LIGHT signal exerts more complex effects on other cells^61^, including inducing HIF2α and Cyclin D1 expression in tumor cells^62^ and promoting inflammatory responses in adipose tissue by combining with HVEM^63^. The evidence presented suggests a potential correlation between TNFRSF14 and TNFSF14 with poorer prognosis in glioblastoma. However, further research is required to establish these associations conclusively.

CD40, a member of the TNFR superfamily, exhibits distinct effects on different cell types. It is ubiquitously expressed on the surface of APCs. CD40 plays a significant role in various essential functions, including B-cell proliferation, differentiation, production of high-affinity antibodies, isotype switching, memory response, and costimulatory activity^64,65^. Furthermore, the interaction between CD40 and its ligand CD40L plays a critical role in controlling the maturation, survival, cytokine production, costimulatory activity, and antimicrobial function of mast cells (MCs), macrophages, DCs, and neutrophils^66^. Interestingly, CD40 regulates endothelial cell survival, proliferation, migration, and angiogenesis through the activation of the PI3K/Akt signaling pathway^67^.

We confirmed the role of CD276 in promoting tumor progression and reducing survival outcomes in glioma through immunohistochemical analysis. CD276, also known as B7-H3, contributes to an immunosuppressive microenvironment characterized by increased secretion of IL-10 and TGF-β1^68^, along with the inhibition of CD4^ +^ T cells, CD8^ +^ T cells, NK cells, macrophages, neutrophils, and DCs. This immunosuppressive effect is also accompanied by the suppression of IFN-γ, IL-2, perforin, and granzyme B secretion^69^. B7-H3 plays a crucial role in regulating the differentiation of TAMs, promoting polarization towards the type 2 phenotype and facilitating the transition from an M1 to an M2 phenotype^70^. Additionally, the expression of B7-H3 is positively correlated with FOXP3 + regulatory T cells, contributing to the establishment of an immunosuppressive microenvironment within the tumor^71^. B7-H3 exerts an inhibitory effect on the activity of NK cells, impairing their ability to induce cell lysis in neuroblastoma and glioma cell lines^72,73^. Consistent with our IHC findings, elevated levels of CD276 expression were found in glioblastoma, which might be associated with its role in facilitating immune evasion^74,75^.

In conclusion, our model can be employed in predicting patient outcomes and response to immune checkpoint blockade in glioblastoma. It also provides insights into the role of disulfidptosis and immune crosstalk, as well as the potential involvement of inflammatory cytokines, immune cell infiltration, angiogenesis, ECM remodeling, and EMT in glioblastoma progression.

## Methods

### Data source and gene compilation

Transcriptomic data and clinicopathological information were acquired from The Cancer Genome Atlas (TCGA) database (https://portal.gdc.cancer.gov/), encompassing a total of 154 patients with glioblastoma. In addition, expression profiles of Chinese Glioma Genome Atlas (CGGA) comprising 657 cases with available overall survival (OS) data were also obtained (http://www.cgga.org.cn/). In the CGGA cohort, there were 249 cases of primary and recurrent glioblastoma. GSE13041 (GPL570) were downloaded from https://www.ncbi.nlm.nih.gov/geo/. The disulfidptosis-related genes were collected from^3^, and the immune checkpoint genes were obtained from^76^.

### Construction and validation of a disulfidptosis related immune checkpoint prognostic signature

The correlation between disulfidptosis-related genes and immune checkpoint genes was explored within the expression profile datasets from TCGA and was calculated using the "cor.test" function in R. DRIC genes with an absolute correlation coefficient > 0.3 and *p* value < 0.05 were utilized for univariate Cox regression analyses. The R packages "survival" and "survminer" were employed to identify nine DRIC genes that exhibited significant associations with OS through univariate Cox regression analyses. Hazard ratios (HRs) and 95% confidence intervals (CIs) were calculated for these 9 DRIC genes, and a forest plot was constructed to visualize the results. To address multicollinearity in gene expression analysis, the the least absolute shrinkage and delection operator (LASSO)-Cox method was employed. Then, a prognostic model was developed for glioblastoma by incorporating the expression levels of six DRIC genes, utilizing the following formula:

$$risk\;score\;{ }\left( x \right) = \mathop \sum \limits_{x}^{n} \left( {Exp(x)*{\text{Coef}}({\text{x)}}} \right).$$

Exp(x) stands for the mRNA level of each gene in the prognostic model, and the regression coefficient (Coef(x)) represents the specific coefficient assigned to each gene in the model. Subsequently, the risk score was calculated utilizing the same formula in the CGGA cohort. Based on the median value of the risk score, the cohorts were stratified into high- and low-risk groups. The comparison of OS between these groups was conducted using Kaplan–Meier analysis, implemented with the R package "survival". Furthermore, the R package "timeROC" was employed for assessing the operating characteristic curve (ROC) and calculating the area under the curve (AUC) to compare 1-year, 3-year, 4-year, and 5-year survival rates between the high-risk and low-risk groups. A univariate Cox regression analysis was conducted to examine the influence of sex, race, age, risk score, and IDH status on the survival outcome for OS. HRs along with their corresponding 95% CIs were utilized to assess the significance of these variables. Furthermore, a multivariate Cox regression analysis was performed to assess the independent impact of the risk score on OS, while adjusting for other relevant variables. A user-friendly and clinically adaptable nomogram based on the risk score was then constructed with additional clinicopathologic features including race, isocitrate dehydrogenase (IDH) status, and age, utilizing the "rms" package in R. This nomogram enabled the estimation of OS probabilities for patients with glioblastoma at 0.5 years, 1 year, and 2 years. To validate the nomogram-based prediction model, calibration curves across 0.5, 1, and 2 years with 1000 bootstrap resamplings were generated utilizing the "rms" package in R. Furthermore, decision curve analysis (DCA) was performed utilizing the "stdca" function in R to assess the clinical utility of nomograms in guiding clinical decision-making^77^.

### Detection of differentially expressed genes and analysis of functional enrichment

The identification of differentially expressed genes (DEGs) for the high-risk and low-risk groups was performed utilizing the "limma" package in R. DEGs were identified based on a significance threshold of adjusted *p* value < 0.05 and the absolute value of log2 fold change > 1.5. Volcano plots were generated to visually represent the DEGs. To explore the functional implications of the risk score, an assessment of Gene Ontology (GO) terms and Kyoto Encyclopedia of Genes and Genomes (KEGG) pathways was performed using the "GSEA" package in R. Additionally, gene set variation analysis (GSVA) was conducted using the "GSVA" package in R. The gene set variation analysis (GSVA) was conducted using the C2: curated gene sets and C7: immunologic signature gene sets from the MsigDB as reference molecular signature databases. Statistical significance was determined at an adjusted *p* value < 0.05. Additionally, the single-sample gene set enrichment analysis (ssGSEA) was employed to assess both the disulfidptosis score and the score of tumor-associated macrophages (TAM). The TAM-related genes were obtained from^19^.

### Discovery of hub genes and regulatory networks

The "GOSemSim" package was utilized to determine the central genes within the group of DEGs. Protein–protein interactions (PPIs) among the DEGs were investigated using the STRING database (https://cn.string-db.org/).

### Analysis of immune infiltration

To assess the TME, the Immuno-Oncology Biological Research (IOBR) package^78^ was used which includes the algorithms CIBERSORT, MCPCOUNTER, EPIC, xCELL, ESTIMATE, quanTIseq, TIMER, and IPS. Subsequently, the association between the components and scoring of the TME with the risk score and OS was evaluated by the Pearson method.

### Immunohistochemistry analysis

Immunohistochemistry analysis (IHC) assays were performed to assess the expression of CD276 using a Rabbit monoclonal [EPR20115] antibody against CD276 (1:80,000, Abcam, ab219648) on 144 cases of glioma pathological sections. The assays were conducted using a biotin assay system (Beijing Zhongshan Jinqiao, China, Cat. No. PV-9001, PV-9002), following the manufacturer's instructions. For IHC analysis, the grading system utilized the following criteria: absence of staining denoted a score of 0, yellow staining denoted a score of 1, and brown staining denoted a score of 2. Based on the percentage of positively stained tumor cells in the visual field, a score of 0 was assigned for < 1% cells, a score of 1 for 1–25%, a score of 2 for 25–75%, and a score of 3 for 75–100%. The overall score was determined as the product of the intensity score and the percentage of positive cells.

### Statistical analysis

The data analysis was performed using R statistical software (version 4.1.3). Descriptive statistics, including the mean and standard deviation, were computed for continuous variables. For comparing means between two groups, Student's t-test was applied, while one-way analysis of variance (ANOVA) followed by post hoc Tukey's test was used for multiple group comparisons. Logistic regression analysis was conducted to assess the association between categorical variables, with adjustments made for potential confounders including age, sex, and disease stage. A p-value of less than 0.05 was considered statistically significant, and all tests were two-sided.

### Supplementary Information

Supplementary Information.

## Data Availability

The datasets used and/or analysed during the current study available from the corresponding author on reasonable request.
