# Supplementary material for: Cross-talk between disulfidptosis and immune check point genes defines the tumor microenvironment for the prediction of prognosis and immunotherapies in glioblastoma
Source: Sci Rep. 2024 Feb 16;14:3901. doi: 10.1038/s41598-024-52128-x (PMC10873294; doi:10.1038/s41598-024-52128-x)
Supplement: Supplementary file 1 — Supplementary Information. [file 41598_2024_52128_MOESM1_ESM.docx]

Supplementary Table 1. The correlation between disulfidptosis genes and ICGs.

| Disulfidptosis genes | ICGs | coefficient | | p value | | correlation |
| --- | --- | --- | --- | --- | --- | --- |
| FLNA | BTN2A2 | 0.383721664081285 | | 8.99490660623682e-07 | | positive |
| MYH9 | BTN2A2 | 0.343538225684171 | | 1.28897457441917e-05 | | positive |
| TLN1 | BTN2A2 | 0.43232938944738 | | 2.15558254442685e-08 | | positive |
| MYL6 | BTN2A2 | -0.354761372376679 | | 6.35200946563605e-06 | | negative |
| MYH10 | BTN2A2 | 0.473374683521171 | | 5.6462348023348e-10 | | positive |
| IQGAP1 | BTN2A2 | 0.332302517642598 | | 2.54861534030089e-05 | | positive |
| CD2AP | BTN2A2 | 0.486041650453275 | | 1.65889735121298e-10 | | positive |
| SLC7A11 | BTN2A2 | 0.300905865567223 | | 0.000149446195206674 | | positive |
| CD2AP | CEACAM1 | 0.312707138282148 | | 7.86541323190094e-05 | | positive |
| PDLIM1 | TDO2 | 0.311854941789089 | | 8.24609224967248e-05 | | positive |
| FLNB | VTCN1 | 0.360704335465356 | | 4.31882070177838e-06 | | positive |
| FLNB | ADORA2A | 0.34714926547286 | | 1.02953360500421e-05 | | positive |
| MYL6 | ADORA2A | -0.435109408712305 | | 1.70967629220893e-08 | | negative |
| MYH10 | ADORA2A | 0.422069783172117 | | 4.98050196593387e-08 | | positive |
| DSTN | ADORA2A | -0.344184945580949 | | 1.23837598753444e-05 | | negative |
| INF2 | ADORA2A | 0.320697639663692 | | 5.01429268603921e-05 | | positive |
| FLNA | CD276 | 0.66077545070213 | | 1.11518261973716e-20 | | positive |
| MYH9 | CD276 | 0.605580382709409 | | 8.79574597437204e-17 | | positive |
| TLN1 | CD276 | 0.435153105840756 | | 1.70343120941722e-08 | | positive |
| ACTB | CD276 | 0.608227863956284 | | 5.95010467490681e-17 | | positive |
| MYH10 | CD276 | 0.329416126364543 | | 3.02357634150612e-05 | | positive |
| IQGAP1 | CD276 | 0.549728036599746 | | 1.53949777614072e-13 | | positive |
| ACTN4 | CD276 | 0.666160409830789 | | 4.19384080343355e-21 | | positive |
| PDLIM1 | CD276 | 0.34733670542962 | | 1.0175158673674e-05 | | positive |
| INF2 | CD276 | 0.389059187591805 | | 6.14336173332522e-07 | | positive |
| MYH9 | CD274 | 0.365067155883358 | | 3.23751693112232e-06 | | positive |
| TLN1 | CD274 | 0.306137675357482 | | 0.000112812277123529 | | positive |
| ACTB | CD274 | 0.335470540207052 | | 2.10856024723585e-05 | | positive |
| IQGAP1 | CD274 | 0.581287046466837 | | 2.69780809641168e-15 | | positive |
| ACTN4 | CD274 | 0.306462716333311 | | 0.00011083902538075 | | positive |
| CD2AP | CD274 | 0.452894263410033 | | 3.69135914544727e-09 | | positive |
| CAPZB | PDCD1LG2 | 0.31853328587291 | | 5.67170434622682e-05 | | positive |
| IQGAP1 | PDCD1LG2 | 0.515107131440712 | | 8.19769282190939e-12 | | positive |
| CD2AP | PDCD1LG2 | 0.566403865112651 | | 1.91678109270403e-14 | | positive |
| MYH9 | CD28 | 0.317594643757462 | | 5.9812315208703e-05 | | positive |
| CAPZB | CD28 | 0.316407972886915 | | 6.39523091945067e-05 | | positive |
| CD2AP | CD28 | 0.301905227285412 | | 0.000141688273783308 | | positive |
| CAPZB | CD80 | 0.399874035538827 | | 2.77860309357191e-07 | | positive |
| CD2AP | CD80 | 0.373385452672923 | | 1.84695381359201e-06 | | positive |
| MYH10 | CD86 | -0.371813712695199 | | 2.0560548394676e-06 | | negative |
| CAPZB | CD86 | 0.506411288109015 | | 2.0770166004425e-11 | | positive |
| CD2AP | CD86 | 0.36993348067301 | | 2.3357981848567e-06 | | positive |
| FLNA | CD160 | 0.320880698176981 | | 4.96210155180833e-05 | | positive |
| MYL6 | CD160 | -0.358969766825619 | | 4.83743145638783e-06 | | negative |
| MYH10 | CD160 | 0.323871787302742 | | 4.17832994904267e-05 | | positive |
| IQGAP1 | TNFRSF14 | 0.395900582717392 | | 3.73123843515441e-07 | | positive |
| CD2AP | TNFRSF14 | 0.445188510943643 | | 7.24979173990503e-09 | | positive |
| CD2AP | TNFSF14 | 0.358548580745035 | | 4.97198872806511e-06 | | positive |
| MYH9 | TNFRSF9 | 0.380503329429493 | | 1.12833432889034e-06 | | positive |
| CAPZB | TNFRSF9 | 0.318542514755528 | | 5.66873698070142e-05 | | positive |
| IQGAP1 | TNFRSF9 | 0.307365224994803 | | 0.000105527797194398 | | positive |
| PDLIM1 | TNFRSF9 | 0.314477674820841 | | 7.12649144147401e-05 | | positive |
| CD2AP | TNFRSF9 | 0.390365833737501 | | 5.59013587818028e-07 | | positive |
| MYH9 | TNFSF4 | 0.359759661715414 | | 4.59433915165484e-06 | | positive |
| IQGAP1 | TNFSF4 | 0.449437888490488 | | 5.00695137442999e-09 | | positive |
| CD2AP | TNFSF4 | 0.430384303232979 | | 2.53192277816761e-08 | | positive |
| CD2AP | CD70 | 0.319883617489231 | | 5.25267585067661e-05 | | positive |
| MYH9 | CD40 | 0.366927702695321 | | 2.85942866235445e-06 | | positive |
| CAPZB | CD40 | 0.425311277430579 | | 3.83410682077628e-08 | | positive |
| PDLIM1 | CD40 | 0.451307613229916 | | 4.24758204541868e-09 | | positive |
| MYH10 | HAVCR2 | -0.353617840473453 | | 6.8354197973552e-06 | | negative |
| CAPZB | HAVCR2 | 0.510425152374204 | | 1.35673037651609e-11 | | positive |
| TLN1 | LGALS9 | 0.30053065613069 | | 0.000152459783554772 | | positive |
| CAPZB | LGALS9 | 0.461219893331616 | | 1.74653267467803e-09 | | positive |
| MYH9 | TNFRSF18 | 0.377141162973415 | | 1.42613936610844e-06 | | positive |
| IQGAP1 | TNFRSF18 | 0.328381508039514 | | 3.21323683971959e-05 | | positive |
| CAPZB | CD47 | 0.324656381984245 | | 3.99289407761969e-05 | | positive |
| IQGAP1 | CD47 | 0.367517327774795 | | 2.74863522264022e-06 | | positive |
| MYH9 | SIRPA | 0.440073580835259 | | 1.12440116110444e-08 | | positive |
| TLN1 | SIRPA | 0.474696959129635 | | 4.98013189741334e-10 | | positive |
| MYL6 | SIRPA | -0.359166526246816 | | 4.77576239389799e-06 | | negative |
| IQGAP1 | SIRPA | 0.36870784704754 | | 2.53723838605367e-06 | | positive |
| ACTN4 | SIRPA | 0.449966618122979 | | 4.77988277630575e-09 | | positive |
| CD2AP | SIRPA | 0.384721610195169 | | 8.37908212344431e-07 | | positive |
| INF2 | SIRPA | 0.30230181914435 | | 0.000138714986593381 | | positive |
| SLC7A11 | SIRPA | 0.471823249711743 | | 6.53777401903247e-10 | | positive |
| CAPZB | CD226 | 0.397063856923664 | | 3.42408414056759e-07 | | positive |
| CD2AP | CD226 | 0.373620776831307 | | 1.81744440879816e-06 | | positive |
| CD2AP | CD96 | 0.36177139555408 | | 4.02647232416612e-06 | | positive |
| MYH9 | TIGIT | 0.301897039984762 | | 0.00014175027620411 | | positive |
| TLN1 | TIGIT | 0.330242385559303 | | 2.87972945749832e-05 | | positive |
| CD2AP | TIGIT | 0.433509369770061 | | 1.95413193333826e-08 | | positive |
| FLNA | PVR | 0.672705410009403 | | 1.24268444632585e-21 | | positive |
| FLNB | PVR | 0.304147764722235 | | 0.000125625861818581 | | positive |
| MYH9 | PVR | 0.679986015906761 | | 3.09496138000516e-22 | | positive |
| TLN1 | PVR | 0.531691238168588 | | 1.29188916522823e-12 | | positive |
| ACTB | PVR | 0.512258164363552 | | 1.11488092203411e-11 | | positive |
| MYH10 | PVR | 0.383055590044577 | | 9.42880149412407e-07 | | positive |
| IQGAP1 | PVR | 0.550502723972893 | | 1.40104247967902e-13 | | positive |
| ACTN4 | PVR | 0.770944342481355 | | 1.38187224803958e-31 | | positive |
| PDLIM1 | PVR | 0.311237292421736 | | 8.53270013802304e-05 | | positive |
| CD2AP | PVR | 0.353028901611261 | | 7.09777850227841e-06 | | positive |
| INF2 | PVR | 0.454852782390786 | | 3.10108318298916e-09 | | positive |
| FLNA | BTN2A1 | 0.591875275340247 | | 6.2854757941956e-16 | | positive |
| FLNB | BTN2A1 | 0.408509221432146 | | 1.44472695223027e-07 | | positive |
| MYH9 | BTN2A1 | 0.484181520701208 | | 1.99205764078956e-10 | | positive |
| TLN1 | BTN2A1 | 0.513016210666208 | | 1.02758582046767e-11 | | positive |
| MYL6 | BTN2A1 | -0.379409777668079 | | 1.21800345891657e-06 | | negative |
| MYH10 | BTN2A1 | 0.547297724554523 | | 2.06583014289969e-13 | | positive |
| ACTN4 | BTN2A1 | 0.588635895722853 | | 9.87070181316389e-16 | | positive |
| CD2AP | BTN2A1 | 0.343982543550781 | | 1.25400634879398e-05 | | positive |
| INF2 | BTN2A1 | 0.484791827863783 | | 1.87618800635561e-10 | | positive |
| FLNB | CD209 | 0.348518705290552 | | 9.44733743887984e-06 | | positive |
| MYH9 | CD209 | 0.379055941383577 | | 1.24844264656656e-06 | | positive |
| CAPZB | CD209 | 0.302673058197181 | | 0.000135984593374635 | | positive |
| PDLIM1 | CD209 | 0.30258283641874 | | 0.000136643507882417 | | positive |
| CD2AP | CD209 | 0.359933775861852 | | 4.5423361248384e-06 | | positive |
| MYH9 | KIR2DL3 | 0.309899007399248 | | 9.18597690207693e-05 | | positive |
| CAPZB | KIR2DL4 | 0.361861437318566 | | 4.00268165743327e-06 | | positive |
| CD2AP | KIR2DL4 | 0.347968353967186 | | 9.77986906586461e-06 | | positive |
| ACTB | HLA-A | 0.325843799383697 | | 3.72689971541248e-05 | | positive |
| MYL6 | HLA-A | 0.325931696791621 | | 3.70788685910443e-05 | | positive |
| IQGAP1 | HLA-B | 0.353852120750254 | | 6.73362882357763e-06 | | positive |
| CD2AP | HLA-B | 0.320151934005653 | | 5.17295005250418e-05 | | positive |
| IQGAP1 | HLA-C | 0.309843371020213 | | 9.2141226416819e-05 | | positive |
| MYH10 | HLA-DMA | -0.3986438975241 | | 3.04537392298807e-07 | | negative |
| CAPZB | HLA-DMA | 0.412616165966048 | | 1.05161794089456e-07 | | positive |
| CD2AP | HLA-DMA | 0.307724651358949 | | 0.000103479563781817 | | positive |
| MYL6 | HLA-DMB | 0.302772822494004 | | 0.000135259438149789 | | positive |
| MYH10 | HLA-DMB | -0.503266244670143 | | 2.88854901642347e-11 | | negative |
| CAPZB | HLA-DMB | 0.365642035235576 | | 3.11590069998721e-06 | | positive |
| ACTN4 | HLA-DMB | -0.358205994734345 | | 5.0840447785695e-06 | | negative |
| CAPZB | HLA-DOA | 0.41246850685774 | | 1.06377317752116e-07 | | positive |
| CD2AP | HLA-DOA | 0.391984954129502 | | 4.97041060081923e-07 | | positive |
| CAPZB | HLA-DPA1 | | 0.370755206272346 | | 2.20935839283014e-06 | positive |
| CD2AP | HLA-DPA1 | | 0.32647613393079 | | 3.59213727795146e-05 | positive |
| MYH10 | HLA-DPB1 | | -0.322649509969953 | | 4.48340248883513e-05 | negative |
| CAPZB | HLA-DPB1 | | 0.376325149452772 | | 1.50896772634511e-06 | positive |
| CAPZB | HLA-DQA1 | | 0.374982891179584 | | 1.65525515059845e-06 | positive |
| CD2AP | HLA-DQA1 | | 0.365579117444053 | | 3.12899649051872e-06 | positive |
| CAPZB | HLA-DQB1 | | 0.393644612053173 | | 4.40353498828773e-07 | positive |
| CD2AP | HLA-DQB1 | | 0.313317501098347 | | 7.60294075135375e-05 | positive |
| MYL6 | HLA-DRA | | 0.34549185922339 | | 1.14179660257971e-05 | positive |
| MYH10 | HLA-DRA | | -0.475189513835088 | | 4.75196368840128e-10 | negative |
| CAPZB | HLA-DRA | | 0.37364014141981 | | 1.81503618641021e-06 | positive |
| MYH10 | HLA-DRB1 | | -0.37190587517629 | | 2.04319675172985e-06 | negative |
| CAPZB | HLA-DRB1 | | 0.395046984643609 | | 3.97318785134856e-07 | positive |
| CD2AP | HLA-DRB1 | | 0.302722316997254 | | 0.000135626094692131 | positive |
| MYH9 | HLA-E | | 0.319718928150922 | | 5.30217995186909e-05 | positive |
| CAPZB | HLA-E | | 0.328066366498786 | | 3.27319459595696e-05 | positive |
| IQGAP1 | HLA-E | | 0.395250235043458 | | 3.91425343150332e-07 | positive |
| CD2AP | HLA-E | | 0.436790095454406 | | 1.48465098602168e-08 | positive |
| CD2AP | HLA-F | | 0.317487522457071 | | 6.01754558341745e-05 | positive |
| MYL6 | LAG3 | | -0.330869420977778 | | 2.77489154488011e-05 | negative |

Supplementary Table 2. The LASSO genes and corresponding coefficients

| Gene | Coef |
| --- | --- |
| CD276 | 0.175444040407167 |
| TNFRSF14 | 0.0318195390182175 |
| TNFSF14 | 0.119667406337046 |
| TNFSF4 | 0.198775061150048 |
| CD40 | 0.0680303339698873 |
| TNFRSF18 | 0.0362716045149574 |

Supplementary Table 3. Fold Change of Differential Expression Genes between High and Low-Risk Groups

| Gene | logFC | AveExpr | t | P.Value | adj.P.Val | B | change |
| --- | --- | --- | --- | --- | --- | --- | --- |
| HES5 | -2.24023 | 1.163453 | -7.59619 | 2.79E-12 | 1.28E-09 | 17.16866 | DOWN |
| GSX1 | -1.82202 | 1.28558 | -4.84487 | 3.07E-06 | 6.4E-05 | 4.31601 | DOWN |
| SIX6 | -1.82043 | -1.76082 | -3.82677 | 0.000189 | 0.001671 | 0.596335 | DOWN |
| FREM3 | -1.79126 | -2.03504 | -4.37685 | 2.22E-05 | 0.000309 | 2.507598 | DOWN |
| SMIM18 | -1.73672 | -0.6862 | -4.9512 | 1.92E-06 | 4.45E-05 | 4.70411 | DOWN |
| KCTD4 | -1.68583 | 0.169037 | -5.96251 | 1.64E-08 | 1.18E-06 | 9.059574 | DOWN |
| BTBD17 | -1.57643 | 3.200949 | -5.55652 | 1.19E-07 | 5.34E-06 | 7.339141 | DOWN |
| DLX6 | -1.55746 | -0.7652 | -4.28469 | 3.22E-05 | 0.000421 | 2.171651 | DOWN |
| RPRM | -1.53529 | 2.059595 | -4.7322 | 5E-06 | 9.58E-05 | 3.864263 | DOWN |
| ZNF560 | -1.50484 | -1.75387 | -4.17164 | 5.04E-05 | 0.000602 | 1.770345 | DOWN |
| ASCL1 | -1.50071 | 5.115427 | -6.11273 | 7.75E-09 | 6.48E-07 | 9.84536 | DOWN |
| CCL18 | 1.519661 | -0.40938 | 3.407775 | 0.000836 | 0.00523 | -0.7177 | UP |
| LRRC15 | 1.524519 | -0.66947 | 4.23375 | 3.94E-05 | 0.000493 | 1.989942 | UP |
| MMP9 | 1.531123 | 4.18613 | 4.762719 | 4.38E-06 | 8.59E-05 | 3.85435 | UP |
| BDKRB2 | 1.552496 | 2.135002 | 6.631214 | 5.35E-10 | 8.07E-08 | 12.39479 | UP |
| AHNAK2 | 1.572823 | 3.378085 | 5.513859 | 1.45E-07 | 6.23E-06 | 7.141128 | UP |
| MMP1 | 1.605537 | -0.78119 | 4.720607 | 5.26E-06 | 9.91E-05 | 3.796946 | UP |
| MXRA5 | 1.606088 | 4.451971 | 6.829431 | 1.87E-10 | 3.62E-08 | 13.47036 | UP |
| TREML2 | 1.64162 | -1.91299 | 7.040352 | 6E-11 | 1.4E-08 | 14.13443 | UP |
| THBS1 | 1.706301 | 5.439016 | 6.499224 | 1.07E-09 | 1.39E-07 | 11.74937 | UP |
| FPR2 | 1.71524 | 0.580465 | 6.645999 | 4.95E-10 | 7.65E-08 | 12.30344 | UP |
| CXCL3 | 1.782684 | 1.041283 | 6.163457 | 6E-09 | 5.46E-07 | 10.04948 | UP |
| EREG | 1.806742 | -1.7608 | 5.300772 | 3.94E-07 | 1.33E-05 | 6.141706 | UP |
| IBSP | 1.834289 | 2.989986 | 5.030247 | 1.35E-06 | 3.42E-05 | 5.055652 | UP |
| PTX3 | 1.887288 | 4.408598 | 7.309119 | 1.38E-11 | 4.37E-09 | 15.98599 | UP |
| LIF | 1.907508 | 3.632922 | 6.738726 | 3.03E-10 | 5.41E-08 | 13.01449 | UP |
| CXCL5 | 1.975301 | 0.693788 | 5.7462 | 4.76E-08 | 2.64E-06 | 8.119514 | UP |
| CD300E | 2.177327 | -0.04952 | 8.728336 | 4.06E-15 | 1.49E-11 | 22.94924 | UP |
| MARCO | 2.291578 | 1.09691 | 6.031485 | 1.16E-08 | 8.68E-07 | 9.443397 | UP |
| PI3 | 2.499875 | 1.751929 | 5.780571 | 4.03E-08 | 2.34E-06 | 8.334712 | UP |

Supplementary Table 4. The association between infiltrating cells and risk score on overall survival.

| ID | Hazard_ratio | Low_CI | Ligh_CI | p_value | weight | weight_HR |
| --- | --- | --- | --- | --- | --- | --- |
| riskScore | 3.427395 | 1.990577 | 5.901324 | 8.87E-06 | 5.05225 | 242.739505 |
| B_cells_naive_CIBERSORT | 1.009015 | 0.804626 | 1.265321 | 0.938061 | 0.027769 | 0.90145887 |
| B_cells_memory_CIBERSORT | 0.97879 | 0.787321 | 1.216822 | 0.84694 | 0.072147 | -2.1209771 |
| Plasma_cells_CIBERSORT | 1.027173 | 0.719742 | 1.46592 | 0.882548 | 0.054262 | 2.71728593 |
| T_cells_CD8_CIBERSORT | 0.93233 | 0.750981 | 1.157472 | 0.5255 | 0.279427 | -6.7669663 |
| T_cells_CD4_memory_resting_CIBERSORT | 0.991704 | 0.808509 | 1.216407 | 0.936279 | 0.028595 | -0.8296131 |
| T_cells_CD4_memory_activated_CIBERSORT | 0.997329 | 0.765517 | 1.299337 | 0.984189 | 0.006921 | -0.2671063 |
| T_cells_follicular_helper_CIBERSORT | 1.094051 | 0.887011 | 1.349417 | 0.401024 | 0.396829 | 9.40511048 |
| T_cells_regulatory_(Tregs)_CIBERSORT | 1.034992 | 0.855196 | 1.252588 | 0.723888 | 0.140329 | 3.4991841 |
| T_cells_gamma_delta_CIBERSORT | 1.001617 | 0.699288 | 1.434654 | 0.992968 | 0.003065 | 0.16170463 |
| NK_cells_resting_CIBERSORT | 1.156502 | 0.965214 | 1.385699 | 0.114988 | 0.939349 | 15.6501748 |
| NK_cells_activated_CIBERSORT | 1.02707 | 0.846484 | 1.246182 | 0.786604 | 0.104244 | 2.707001 |
| Monocytes_CIBERSORT | 0.953561 | 0.796802 | 1.141159 | 0.603802 | 0.219105 | -4.6439011 |
| Macrophages_M0_CIBERSORT | 1.144974 | 0.927818 | 1.412954 | 0.207047 | 0.683931 | 14.4973519 |
| Macrophages_M1_CIBERSORT | 0.898913 | 0.696051 | 1.160898 | 0.414124 | 0.38287 | -10.108681 |
| Macrophages_M2_CIBERSORT | 0.884695 | 0.738022 | 1.060517 | 0.185285 | 0.732161 | -11.530503 |
| Dendritic_cells_resting_CIBERSORT | 1.426813 | 1.077451 | 1.889455 | 0.013117 | 1.882155 | 42.6812989 |
| Dendritic_cells_activated_CIBERSORT | 1.03658 | 0.711127 | 1.510978 | 0.85177 | 0.069677 | 3.65796475 |
| Mast_cells_resting_CIBERSORT | 0.978771 | 0.793526 | 1.20726 | 0.841131 | 0.075136 | -2.1228932 |
| Mast_cells_activated_CIBERSORT | 1.036326 | 0.832192 | 1.290533 | 0.749883 | 0.125006 | 3.63258882 |
| Eosinophils_CIBERSORT | 0.875035 | 0.654112 | 1.170575 | 0.368577 | 0.433472 | -12.496474 |
| Neutrophils_CIBERSORT | 0.990136 | 0.798959 | 1.227058 | 0.927838 | 0.032528 | -0.986404 |
| T_cells_MCPcounter | 1.280049 | 1.02797 | 1.593944 | 0.02735 | 1.563051 | 28.0049097 |
| CD8_T_cells_MCPcounter | 1.155353 | 0.927352 | 1.43941 | 0.197916 | 0.703518 | 15.5353029 |
| Cytotoxic_lymphocytes_MCPcounter | 1.043518 | 0.859315 | 1.267207 | 0.667281 | 0.175691 | 4.35184477 |
| B_lineage_MCPcounter | 1.223071 | 0.989758 | 1.511383 | 0.062233 | 1.205978 | 22.3070992 |
| NK_cells_MCPcounter | 1.164694 | 0.937688 | 1.446658 | 0.168108 | 0.774412 | 16.4694464 |
| Monocytic_lineage_MCPcounter | 1.115298 | 0.926705 | 1.342272 | 0.248265 | 0.605085 | 11.5298308 |
| Myeloid_dendritic_cells_MCPcounter | 1.174695 | 0.947473 | 1.456408 | 0.142102 | 0.847399 | 17.4694557 |
| Neutrophils_MCPcounter | 1.123862 | 0.917285 | 1.376961 | 0.259816 | 0.585333 | 12.3862081 |
| Endothelial_cells_MCPcounter | 1.001536 | 0.826721 | 1.213317 | 0.987484 | 0.00547 | 0.15364513 |
| Fibroblasts_MCPcounter | 1.179682 | 0.969928 | 1.434796 | 0.098068 | 1.008473 | 17.968188 |
| aDC_xCell | 1.698259 | 0.998413 | 2.888668 | 0.050689 | 1.295087 | 69.8259101 |
| Adipocytes_xCell | 1.239044 | 0.957111 | 1.604023 | 0.103698 | 0.984228 | 23.9043508 |
| Astrocytes_xCell | 1.137831 | 0.83192 | 1.55623 | 0.418983 | 0.377804 | 13.7830949 |
| B-cells_xCell | 1.043764 | 0.812377 | 1.341058 | 0.737646 | 0.132152 | 4.37643164 |
| Basophils_xCell | 0.903109 | 0.744324 | 1.095767 | 0.301613 | 0.520549 | -9.6890936 |
| CD4+_memory_T-cells_xCell | 1.098313 | 0.872511 | 1.382552 | 0.424535 | 0.372086 | 9.83132862 |
| CD4+_naive_T-cells_xCell | 1.787831 | 1.0385 | 3.077841 | 0.036059 | 1.44299 | 78.7830506 |
| CD4+_T-cells_xCell | 0.844634 | 0.494653 | 1.442236 | 0.536225 | 0.270653 | -15.53661 |
| CD4+_Tcm_xCell | 0.990956 | 0.7967 | 1.232576 | 0.934954 | 0.02921 | -0.9044391 |
| CD4+_Tem_xCell | 1.145851 | 0.951399 | 1.380046 | 0.151321 | 0.8201 | 14.585081 |
| CD8+_naive_T-cells_xCell | 0.793724 | 0.646331 | 0.974729 | 0.027512 | 1.560474 | -20.627574 |
| CD8+_T-cells_xCell | 0.999265 | 0.591059 | 1.689393 | 0.997812 | 9.51E-04 | -0.0734549 |
| CD8+_Tcm_xCell | 1.188802 | 0.86864 | 1.626968 | 0.280009 | 0.552827 | 18.8801839 |
| CD8+_Tem_xCell | 2.046368 | 1.24345 | 3.367742 | 0.004844 | 2.314762 | 104.636755 |
| cDC_xCell | 1.147308 | 0.856227 | 1.537342 | 0.35738 | 0.44687 | 14.7307565 |
| Chondrocytes_xCell | 1.084279 | 0.802688 | 1.464655 | 0.597919 | 0.223358 | 8.4278647 |
| Class-switched_memory_B-cells_xCell | 1.227712 | 1.017425 | 1.481462 | 0.032341 | 1.490252 | 22.7711721 |
| CLP_xCell | 0.99018 | 0.809058 | 1.21185 | 0.923726 | 0.034457 | -0.9819705 |
| CMP_xCell | 0.976254 | 0.591474 | 1.611349 | 0.92511 | 0.033807 | -2.3746262 |
| DC_xCell | 1.487374 | 1.104231 | 2.003458 | 0.008992 | 2.046163 | 48.7373502 |
| Endothelial_cells_xCell | 0.982304 | 0.800092 | 1.206013 | 0.864575 | 0.063197 | -1.769556 |
| Eosinophils_xCell | 0.976405 | 0.791637 | 1.204297 | 0.82346 | 0.084357 | -2.3595152 |
| Epithelial_cells_xCell | 1.357284 | 1.108059 | 1.662564 | 0.003165 | 2.49966 | 35.7283576 |
| Erythrocytes_xCell | 1.333483 | 0.842487 | 2.110627 | 0.2193 | 0.658962 | 33.348294 |
| Fibroblasts_xCell | 0.947255 | 0.741629 | 1.209894 | 0.664302 | 0.177634 | -5.2744627 |
| GMP_xCell | 1.071967 | 0.858857 | 1.337958 | 0.538868 | 0.268517 | 7.19672991 |
| Hepatocytes_xCell | 0.895403 | 0.740154 | 1.083217 | 0.255461 | 0.592675 | -10.459667 |
| HSC_xCell | 1.039503 | 0.851428 | 1.269123 | 0.703599 | 0.152675 | 3.95032876 |
| iDC_xCell | 1.2845 | 0.973751 | 1.694418 | 0.076439 | 1.116683 | 28.4499967 |
| Keratinocytes_xCell | 1.466466 | 1.127495 | 1.907347 | 0.004308 | 2.365771 | 46.6466464 |
| ly_Endothelial_cells_xCell | 0.917289 | 0.743369 | 1.131901 | 0.420896 | 0.375825 | -8.2710585 |
| Macrophages_xCell | 1.13544 | 0.930637 | 1.385312 | 0.210708 | 0.676319 | 13.5439669 |
| Macrophages_M1_xCell | 1.151032 | 0.939336 | 1.410439 | 0.174956 | 0.757071 | 15.1032324 |
| Macrophages_M2_xCell | 1.194602 | 0.973929 | 1.465274 | 0.087925 | 1.055888 | 19.4601651 |
| Mast_cells_xCell | 0.861486 | 0.698589 | 1.062368 | 0.163251 | 0.787144 | -13.851377 |
| Megakaryocytes_xCell | 1.013738 | 0.814681 | 1.261431 | 0.902637 | 0.044487 | 1.37375093 |
| Melanocytes_xCell | 1.050104 | 0.866132 | 1.273154 | 0.618839 | 0.208422 | 5.01042699 |
| Memory_B-cells_xCell | 0.901838 | 0.589877 | 1.378782 | 0.633351 | 0.198356 | -9.816152 |
| MEP_xCell | 0.945319 | 0.795768 | 1.122976 | 0.522185 | 0.282176 | -5.4681099 |
| Mesangial_cells_xCell | 1.048196 | 0.835987 | 1.314274 | 0.683396 | 0.165327 | 4.8196341 |
| Monocytes_xCell | 1.195048 | 0.943322 | 1.513947 | 0.139815 | 0.854448 | 19.5047652 |
| MPP_xCell | 1.126873 | 0.907855 | 1.398727 | 0.278693 | 0.554873 | 12.6872521 |
| MSC_xCell | 1.31273 | 1.08866 | 1.582917 | 0.004378 | 2.358707 | 31.2729516 |
| mv_Endothelial_cells_xCell | 0.929882 | 0.758398 | 1.140141 | 0.48457 | 0.314643 | -7.0118159 |
| Myocytes_xCell | 0.966039 | 0.788394 | 1.183711 | 0.738942 | 0.13139 | -3.3961182 |
| naive_B-cells_xCell | 1.58392 | 0.998637 | 2.512226 | 0.050681 | 1.295154 | 58.3919942 |
| Neurons_xCell | 0.946003 | 0.754212 | 1.186565 | 0.631093 | 0.199906 | -5.3997332 |
| Neutrophils_xCell | 1.148359 | 0.782505 | 1.685266 | 0.479677 | 0.319051 | 14.8359091 |
| NK_cells_xCell | 0.999046 | 0.795688 | 1.254377 | 0.993442 | 0.002857 | -0.0953961 |
| NKT_xCell | 1.146545 | 0.951778 | 1.381169 | 0.149963 | 0.824015 | 14.6545237 |
| Osteoblast_xCell | 0.818928 | 0.644934 | 1.039861 | 0.101168 | 0.994956 | -18.107245 |
| pDC_xCell | 1.193683 | 0.923041 | 1.543678 | 0.177165 | 0.751621 | 19.368286 |
| Pericytes_xCell | 0.874616 | 0.713964 | 1.071416 | 0.195736 | 0.708329 | -12.538435 |
| Plasma_cells_xCell | 0.830224 | 0.676564 | 1.018783 | 0.074788 | 1.126169 | -16.97761 |
| Platelets_xCell | 0.902037 | 0.649888 | 1.252017 | 0.537668 | 0.269486 | -9.7962842 |
| Preadipocytes_xCell | 1.084692 | 0.854895 | 1.376259 | 0.503318 | 0.298158 | 8.46918727 |
| pro_B-cells_xCell | 0.852669 | 0.668518 | 1.087548 | 0.199175 | 0.700766 | -14.733064 |
| Sebocytes_xCell | 1.235457 | 0.947019 | 1.611748 | 0.119073 | 0.924186 | 23.5457464 |
| Skeletal_muscle_xCell | 1.034103 | 0.79351 | 1.347644 | 0.803987 | 0.094751 | 3.41033142 |
| Smooth_muscle_xCell | 0.937357 | 0.785667 | 1.118335 | 0.472613 | 0.325495 | -6.2642635 |
| Tgd_cells_xCell | 1.167254 | 0.695069 | 1.96021 | 0.558739 | 0.252791 | 16.7253516 |
| Th1_cells_xCell | 0.894788 | 0.75017 | 1.067285 | 0.216467 | 0.664609 | -10.521213 |
| Th2_cells_xCell | 1.04408 | 0.856284 | 1.273063 | 0.669836 | 0.174032 | 4.40800791 |
| Tregs_xCell | 1.058484 | 0.830226 | 1.349499 | 0.646497 | 0.189434 | 5.84842089 |
| ImmuneScore_xCell | 1.163972 | 0.921186 | 1.470747 | 0.203317 | 0.691827 | 16.3972356 |
| StromaScore_xCell | 0.996358 | 0.790015 | 1.256594 | 0.975414 | 0.010811 | -0.3642221 |
| MicroenvironmentScore_xCell | 1.166801 | 0.919919 | 1.479939 | 0.203438 | 0.691568 | 16.6801073 |
| Bcells_EPIC | 1.391613 | 1.085136 | 1.784649 | 0.009222 | 2.035188 | 39.1613208 |
| CAFs_EPIC | 1.110854 | 0.897965 | 1.374216 | 0.332801 | 0.477816 | 11.0854191 |
| CD4_Tcells_EPIC | 1.199103 | 0.983804 | 1.461519 | 0.072138 | 1.141837 | 19.9102752 |
| CD8_Tcells_EPIC | 1.117613 | 0.898201 | 1.390623 | 0.318681 | 0.496644 | 11.7613173 |
| Endothelial_EPIC | 1.007324 | 0.842457 | 1.204457 | 0.936216 | 0.028624 | 0.73243816 |
| Macrophages_EPIC | 1.073749 | 0.896035 | 1.286711 | 0.440821 | 0.355737 | 7.37492425 |
| NKcells_EPIC | 0.967052 | 0.702787 | 1.330687 | 0.837012 | 0.077269 | -3.2948007 |
| otherCells_EPIC | 0.816187 | 0.663582 | 1.003888 | 0.054452 | 1.263982 | -18.381267 |
| StromalScore_estimate | 1.162941 | 0.955693 | 1.41513 | 0.131705 | 0.880399 | 16.2940542 |
| ImmuneScore_estimate | 1.137499 | 0.94256 | 1.372756 | 0.179205 | 0.746649 | 13.7499411 |
| ESTIMATEScore_estimate | 1.158586 | 0.956843 | 1.402865 | 0.13155 | 0.880908 | 15.858615 |
| TumorPurity_estimate | 0.85499 | 0.702638 | 1.040376 | 0.117664 | 0.929356 | -14.501048 |
| B_cell_TIMER | 0.906346 | 0.761628 | 1.078563 | 0.267913 | 0.572007 | -9.3653617 |
| T_cell_CD4_TIMER | 1.063807 | 0.897852 | 1.260436 | 0.474737 | 0.323547 | 6.38070508 |
| T_cell_CD8_TIMER | 1.224868 | 1.00399 | 1.494339 | 0.045585 | 1.341177 | 22.4868006 |
| Neutrophil_TIMER | 1.192406 | 0.990178 | 1.435935 | 0.063472 | 1.197415 | 19.2405748 |
| Macrophage_TIMER | 1.19619 | 1.001218 | 1.42913 | 0.048453 | 1.314682 | 19.6190023 |
| DC_TIMER | 1.183901 | 0.975809 | 1.436368 | 0.086958 | 1.060692 | 18.3900808 |
| B_cells_quantiseq | 0.945479 | 0.764362 | 1.169511 | 0.605342 | 0.217999 | -5.4521262 |
| Macrophages_M1_quantiseq | 1.11579 | 0.868582 | 1.433356 | 0.391227 | 0.407571 | 11.5790182 |
| Macrophages_M2_quantiseq | 1.009948 | 0.835623 | 1.220641 | 0.918443 | 0.036948 | 0.99480771 |
| Monocytes_quantiseq | 0.954837 | 0.751648 | 1.212954 | 0.705017 | 0.1518 | -4.5162633 |
| Neutrophils_quantiseq | 1.610547 | 0.621179 | 4.175706 | 0.326873 | 0.485621 | 61.054663 |
| NK_cells_quantiseq | 1.015943 | 0.840092 | 1.228603 | 0.870433 | 0.060265 | 1.59426822 |
| T_cells_CD4_quantiseq | 1.22331 | 0.885829 | 1.689366 | 0.221006 | 0.655595 | 22.3310494 |
| T_cells_CD8_quantiseq | 0.821105 | 0.497679 | 1.354716 | 0.440375 | 0.356178 | -17.88949 |
| Tregs_quantiseq | 0.972683 | 0.606134 | 1.560897 | 0.908621 | 0.041617 | -2.7317012 |
| Dendritic_cells_quantiseq | 1.019472 | 0.847723 | 1.226017 | 0.837668 | 0.076928 | 1.94717291 |
| Other_quantiseq | 0.91703 | 0.754432 | 1.114673 | 0.384415 | 0.4152 | -8.296955 |
| MHC_IPS | 1.044191 | 0.868713 | 1.255115 | 0.645048 | 0.190408 | 4.41906904 |
| EC_IPS | 1.285853 | 1.039277 | 1.590931 | 0.020633 | 1.685439 | 28.5853201 |
| SC_IPS | 0.823374 | 0.681157 | 0.995284 | 0.044556 | 1.351098 | -17.662638 |
| CP_IPS | 0.887429 | 0.739087 | 1.065544 | 0.20065 | 0.69756 | -11.257138 |
| AZ_IPS | 0.838972 | 0.678942 | 1.036721 | 0.103951 | 0.983172 | -16.102815 |
| IPS_IPS | 0.841538 | 0.693779 | 1.020767 | 0.079892 | 1.097494 | -15.846178 |

Supplementary Table 5. The Association between the transcription factors and genes

| Transcription factors | Gene | cor | pvalue | Regulation |
| --- | --- | --- | --- | --- |
| ASCL1 | BTBD17 | 0.721987 | 2.48E-18 | positive |
| ASCL1 | DLL1 | 0.730981 | 5.82E-19 | positive |
| ASCL1 | FAM181B | 0.756665 | 6.56E-21 | positive |
| ASCL1 | HES5 | 0.712971 | 1.01E-17 | positive |
| ASCL1 | NRARP | 0.704528 | 3.55E-17 | positive |
| ASCL1 | OLIG2 | 0.736695 | 2.24E-19 | positive |
| ASCL1 | PURG | 0.71083 | 1.39E-17 | positive |
| ASCL1 | SBK1 | 0.731619 | 5.24E-19 | positive |
| ASCL1 | SOX8 | 0.781643 | 4.74E-23 | positive |
| ASCL1 | ZDHHC22 | 0.760266 | 3.35E-21 | positive |
| BATF | ARL11 | 0.800186 | 7.87E-25 | positive |
| BATF | BCL2A1 | 0.811827 | 4.78E-26 | positive |
| BATF | CCR1 | 0.75517 | 8.65E-21 | positive |
| BATF | CCR5 | 0.712789 | 1.03E-17 | positive |
| BATF | CD300C | 0.714531 | 7.93E-18 | positive |
| BATF | CSTA | 0.794125 | 3.15E-24 | positive |
| BATF | FPR2 | 0.719622 | 3.60E-18 | positive |
| BATF | GNA15 | 0.820819 | 4.80E-27 | positive |
| BATF | GPR65 | 0.728515 | 8.71E-19 | positive |
| BATF | GPR84 | 0.744164 | 6.22E-20 | positive |
| BATF | IL10 | 0.724685 | 1.62E-18 | positive |
| BATF | LRG1 | 0.733817 | 3.64E-19 | positive |
| BATF | LRRC25 | 0.802139 | 4.98E-25 | positive |
| BATF | MAFB | 0.737122 | 2.09E-19 | positive |
| BATF | MNDA | 0.777473 | 1.13E-22 | positive |
| BATF | OSTF1 | 0.774216 | 2.19E-22 | positive |
| BATF | PLEK | 0.747108 | 3.71E-20 | positive |
| BATF | PYCARD | 0.798847 | 1.07E-24 | positive |
| BATF | RHOG | 0.761155 | 2.83E-21 | positive |
| BATF | RNASE2 | 0.774324 | 2.15E-22 | positive |
| BATF | SASH3 | 0.729605 | 7.29E-19 | positive |
| BATF | TLR2 | 0.72947 | 7.46E-19 | positive |
| BATF | TNFAIP8 | 0.741512 | 9.86E-20 | positive |
| BATF | TNFAIP8L2 | 0.797737 | 1.39E-24 | positive |
| BHLHE40 | AHNAK2 | 0.701835 | 5.27E-17 | positive |
| BHLHE40 | CLCF1 | 0.736461 | 2.34E-19 | positive |
| BHLHE40 | FAM20C | 0.718558 | 4.25E-18 | positive |
| BHLHE40 | VASN | 0.719509 | 3.67E-18 | positive |
| CEBPA | IGSF6 | 0.706187 | 2.78E-17 | positive |
| CEBPA | KCNK13 | 0.718536 | 4.27E-18 | positive |
| CEBPA | SLC29A3 | 0.764621 | 1.46E-21 | positive |
| CEBPA | SOWAHD | 0.828242 | 6.51E-28 | positive |
| CEBPA | TLR7 | 0.715534 | 6.80E-18 | positive |
| CEBPB | CD300E | 0.723711 | 1.89E-18 | positive |
| CEBPB | CEBPD | 0.749859 | 2.27E-20 | positive |
| CEBPB | FPR2 | 0.703277 | 4.27E-17 | positive |
| CEBPB | GNA15 | 0.747734 | 3.32E-20 | positive |
| CEBPB | IER3 | 0.710015 | 1.57E-17 | positive |
| CEBPB | SOCS3 | 0.725796 | 1.35E-18 | positive |
| CEBPB | THBS1 | 0.715046 | 7.33E-18 | positive |
| E2F1 | CHAF1B | 0.748941 | 2.68E-20 | positive |
| E2F1 | DNAJC9 | 0.705711 | 2.99E-17 | positive |
| E2F1 | E2F2 | 0.804951 | 2.56E-25 | positive |
| E2F1 | ERCC6L | 0.72558 | 1.40E-18 | positive |
| E2F1 | FEN1 | 0.846203 | 3.40E-30 | positive |
| E2F1 | GINS1 | 0.710028 | 1.57E-17 | positive |
| E2F1 | KIF11 | 0.763724 | 1.73E-21 | positive |
| E2F1 | KIF4A | 0.750107 | 2.17E-20 | positive |
| E2F1 | MCM6 | 0.842454 | 1.07E-29 | positive |
| E2F1 | NEIL3 | 0.722158 | 2.42E-18 | positive |
| E2F1 | RMI2 | 0.803726 | 3.43E-25 | positive |
| E2F1 | ZNF367 | 0.818427 | 8.96E-27 | positive |
| EGR1 | EGR3 | 0.742044 | 9.00E-20 | positive |
| EGR1 | IER2 | 0.814413 | 2.50E-26 | positive |
| FOXK1 | ANAPC2 | 0.704649 | 3.49E-17 | positive |
| FOXK1 | ANKRD52 | 0.7987 | 1.11E-24 | positive |
| FOXK1 | AP5B1 | 0.700664 | 6.24E-17 | positive |
| FOXK1 | BMS1 | 0.713202 | 9.71E-18 | positive |
| FOXK1 | CELSR2 | 0.750204 | 2.13E-20 | positive |
| FOXK1 | COX7B | -0.72947 | 7.45E-19 | negative |
| FOXK1 | FUBP3 | 0.700287 | 6.59E-17 | positive |
| FOXK1 | GBF1 | 0.79684 | 1.70E-24 | positive |
| FOXK1 | GIGYF1 | 0.763288 | 1.88E-21 | positive |
| FOXK1 | LMTK2 | 0.751402 | 1.72E-20 | positive |
| FOXK1 | MLLT1 | 0.740975 | 1.08E-19 | positive |
| FOXK1 | MSL2 | 0.711006 | 1.35E-17 | positive |
| FOXK1 | MTF1 | 0.707426 | 2.32E-17 | positive |
| FOXK1 | NACC1 | 0.78348 | 3.22E-23 | positive |
| FOXK1 | OTUD3 | 0.733245 | 4.00E-19 | positive |
| FOXK1 | P2RY11 | 0.700886 | 6.04E-17 | positive |
| FOXK1 | PATL1 | 0.740865 | 1.10E-19 | positive |
| FOXK1 | PLXNA3 | 0.767321 | 8.64E-22 | positive |
| FOXK1 | PPP1R9B | 0.774615 | 2.02E-22 | positive |
| FOXK1 | RBM15B | 0.732486 | 4.54E-19 | positive |
| FOXK1 | RRP1B | 0.730861 | 5.94E-19 | positive |
| FOXK1 | SKI | 0.793141 | 3.92E-24 | positive |
| FOXK1 | SMG5 | 0.734062 | 3.49E-19 | positive |
| FOXK1 | TMEM256 | -0.72724 | 1.07E-18 | negative |
| FOXK1 | ZNF319 | 0.709461 | 1.71E-17 | positive |
| FOXK1 | ZNF335 | 0.769019 | 6.19E-22 | positive |
| FOXK1 | ZNF629 | 0.782562 | 3.91E-23 | positive |
| FOXK1 | ZNF646 | 0.768584 | 6.74E-22 | positive |
| FOXK1 | ZNF70 | 0.721648 | 2.62E-18 | positive |
| FOXK1 | ZNF805 | 0.720174 | 3.31E-18 | positive |
| FOXK1 | ZNF865 | 0.736928 | 2.16E-19 | positive |
| HOXC11 | HOXC10 | 0.831792 | 2.42E-28 | positive |
| HOXC11 | HOXC13 | 0.871266 | 6.40E-34 | positive |
| HOXC11 | HOXC8 | 0.730164 | 6.66E-19 | positive |
| HOXC9 | HOXC10 | 0.817574 | 1.12E-26 | positive |
| HOXC9 | HOXC6 | 0.779784 | 7.00E-23 | positive |
| HOXC9 | HOXC8 | 0.802408 | 4.68E-25 | positive |
| JUNB | CEBPD | 0.742863 | 7.81E-20 | positive |
| JUNB | IER2 | 0.726831 | 1.15E-18 | positive |
| JUNB | OSM | 0.756737 | 6.47E-21 | positive |
| KAT2B | HEPACAM | 0.719178 | 3.86E-18 | positive |
| LYL1 | ARL11 | 0.756262 | 7.07E-21 | positive |
| LYL1 | C3AR1 | 0.773075 | 2.76E-22 | positive |
| LYL1 | CCRL2 | 0.707323 | 2.35E-17 | positive |
| LYL1 | CD300C | 0.735914 | 2.56E-19 | positive |
| LYL1 | CRYBB1 | 0.70012 | 6.75E-17 | positive |
| LYL1 | DEF6 | 0.801626 | 5.62E-25 | positive |
| LYL1 | EBI3 | 0.752651 | 1.37E-20 | positive |
| LYL1 | GIMAP1 | 0.702061 | 5.10E-17 | positive |
| LYL1 | GPR65 | 0.746981 | 3.79E-20 | positive |
| LYL1 | KCNK13 | 0.732458 | 4.56E-19 | positive |
| LYL1 | LRRC25 | 0.708006 | 2.12E-17 | positive |
| LYL1 | MNDA | 0.763095 | 1.95E-21 | positive |
| LYL1 | NRROS | 0.762275 | 2.29E-21 | positive |
| LYL1 | P2RY13 | 0.732225 | 4.74E-19 | positive |
| LYL1 | PLEK | 0.728988 | 8.07E-19 | positive |
| LYL1 | PYCARD | 0.821078 | 4.48E-27 | positive |
| LYL1 | SASH3 | 0.822531 | 3.05E-27 | positive |
| LYL1 | SELPLG | 0.743749 | 6.69E-20 | positive |
| LYL1 | SOWAHD | 0.78922 | 9.34E-24 | positive |
| LYL1 | TLR7 | 0.77453 | 2.06E-22 | positive |
| LYL1 | TMEM119 | 0.753579 | 1.16E-20 | positive |
| LYL1 | TNFAIP8L2 | 0.804641 | 2.76E-25 | positive |
| NOTCH1 | AARS2 | 0.730941 | 5.86E-19 | positive |
| NOTCH1 | ANAPC2 | 0.809552 | 8.40E-26 | positive |
| NOTCH1 | ANKRD52 | 0.722994 | 2.12E-18 | positive |
| NOTCH1 | ARL10 | 0.734472 | 3.26E-19 | positive |
| NOTCH1 | CELSR2 | 0.741216 | 1.04E-19 | positive |
| NOTCH1 | CHAMP1 | 0.793977 | 3.25E-24 | positive |
| NOTCH1 | DCHS1 | 0.745563 | 4.87E-20 | positive |
| NOTCH1 | FGD1 | 0.718804 | 4.10E-18 | positive |
| NOTCH1 | FUBP3 | 0.8222 | 3.33E-27 | positive |
| NOTCH1 | GIGYF1 | 0.710466 | 1.47E-17 | positive |
| NOTCH1 | GPR173 | 0.740154 | 1.25E-19 | positive |
| NOTCH1 | HNRNPA0 | 0.732621 | 4.44E-19 | positive |
| NOTCH1 | HNRNPA1L2 | 0.71722 | 5.24E-18 | positive |
| NOTCH1 | HNRNPUL2 | 0.754032 | 1.07E-20 | positive |
| NOTCH1 | JMY | 0.700861 | 6.06E-17 | positive |
| NOTCH1 | LMTK2 | 0.750702 | 1.95E-20 | positive |
| NOTCH1 | MMP15 | 0.702028 | 5.12E-17 | positive |
| NOTCH1 | MSL2 | 0.782771 | 3.74E-23 | positive |
| NOTCH1 | NACC1 | 0.70404 | 3.82E-17 | positive |
| NOTCH1 | NOVA2 | 0.750436 | 2.05E-20 | positive |
| NOTCH1 | RBM14 | 0.83565 | 8.05E-29 | positive |
| NOTCH1 | RBM15B | 0.776133 | 1.49E-22 | positive |
| NOTCH1 | RRP1B | 0.700854 | 6.07E-17 | positive |
| NOTCH1 | SKI | 0.702232 | 4.97E-17 | positive |
| NOTCH1 | SPATA5 | 0.717359 | 5.13E-18 | positive |
| NOTCH1 | SURF6 | 0.780486 | 6.05E-23 | positive |
| NOTCH1 | TSHZ1 | 0.706566 | 2.63E-17 | positive |
| NOTCH1 | UBQLN4 | 0.728123 | 9.29E-19 | positive |
| NOTCH1 | VANGL2 | 0.775695 | 1.62E-22 | positive |
| NOTCH1 | ZBTB12 | 0.800812 | 6.80E-25 | positive |
| NOTCH1 | ZBTB34 | 0.709331 | 1.74E-17 | positive |
| NOTCH1 | ZBTB39 | 0.824569 | 1.77E-27 | positive |
| NOTCH1 | ZBTB9 | 0.707052 | 2.45E-17 | positive |
| NOTCH1 | ZNF319 | 0.801233 | 6.17E-25 | positive |
| NOTCH1 | ZNF594 | 0.730698 | 6.10E-19 | positive |
| NOTCH1 | ZNF629 | 0.817804 | 1.05E-26 | positive |
| NOTCH1 | ZNF646 | 0.752511 | 1.41E-20 | positive |
| NOTCH1 | ZNF740 | 0.769933 | 5.17E-22 | positive |
| NOTCH1 | ZNF768 | 0.739535 | 1.39E-19 | positive |
| NOTCH1 | ZNF777 | 0.736727 | 2.23E-19 | positive |
| NOTCH1 | ZNF8 | 0.724442 | 1.68E-18 | positive |
| NOTCH1 | ZXDB | 0.7069 | 2.50E-17 | positive |
| RCOR1 | BAG5 | 0.81806 | 9.85E-27 | positive |
| RCOR1 | BMS1 | 0.757037 | 6.12E-21 | positive |
| RCOR1 | MEX3C | 0.703401 | 4.19E-17 | positive |
| RCOR1 | PATL1 | 0.734069 | 3.49E-19 | positive |
| RCOR1 | RBM15B | 0.709025 | 1.82E-17 | positive |
| RCOR1 | SKI | 0.724137 | 1.76E-18 | positive |
| RCOR1 | TOP1 | 0.706424 | 2.69E-17 | positive |
| RCOR1 | ZBTB2 | 0.738597 | 1.63E-19 | positive |
| RCOR1 | ZNF629 | 0.724111 | 1.77E-18 | positive |
| RCOR1 | ZNF805 | 0.754956 | 8.99E-21 | positive |
| RING1 | ZBTB22 | 0.766142 | 1.09E-21 | positive |
| RING1 | ZBTB9 | 0.711176 | 1.32E-17 | positive |
| SMC3 | BMS1 | 0.804187 | 3.07E-25 | positive |
| SMC3 | BRWD3 | 0.770885 | 4.28E-22 | positive |
| SMC3 | CDC5L | 0.753103 | 1.26E-20 | positive |
| SMC3 | DCLRE1B | 0.740036 | 1.27E-19 | positive |
| SMC3 | EIF4EBP2 | 0.761866 | 2.47E-21 | positive |
| SMC3 | EXOC8 | 0.706395 | 2.70E-17 | positive |
| SMC3 | FGD1 | 0.730048 | 6.78E-19 | positive |
| SMC3 | FUBP3 | 0.795139 | 2.50E-24 | positive |
| SMC3 | GBF1 | 0.746167 | 4.38E-20 | positive |
| SMC3 | GEMIN5 | 0.738322 | 1.70E-19 | positive |
| SMC3 | HNRNPA0 | 0.709924 | 1.59E-17 | positive |
| SMC3 | HNRNPH2 | 0.746657 | 4.02E-20 | positive |
| SMC3 | HNRNPUL2 | 0.722722 | 2.21E-18 | positive |
| SMC3 | JMY | 0.759583 | 3.80E-21 | positive |
| SMC3 | JRKL | 0.724369 | 1.70E-18 | positive |
| SMC3 | KIF5B | 0.750049 | 2.19E-20 | positive |
| SMC3 | KLHL15 | 0.741316 | 1.02E-19 | positive |
| SMC3 | LRRC58 | 0.769001 | 6.21E-22 | positive |
| SMC3 | MSL2 | 0.820158 | 5.71E-27 | positive |
| SMC3 | MTF1 | 0.739503 | 1.39E-19 | positive |
| SMC3 | NHLRC2 | 0.741986 | 9.09E-20 | positive |
| SMC3 | NUCKS1 | 0.760343 | 3.30E-21 | positive |
| SMC3 | PATL1 | 0.766466 | 1.02E-21 | positive |
| SMC3 | RBM15B | 0.748524 | 2.88E-20 | positive |
| SMC3 | RBM27 | 0.704843 | 3.39E-17 | positive |
| SMC3 | RGP1 | 0.729879 | 6.97E-19 | positive |
| SMC3 | RNF168 | 0.712774 | 1.04E-17 | positive |
| SMC3 | RNF169 | 0.704454 | 3.59E-17 | positive |
| SMC3 | RRP1B | 0.768518 | 6.83E-22 | positive |
| SMC3 | SASS6 | 0.703268 | 4.27E-17 | positive |
| SMC3 | SMARCA5 | 0.713397 | 9.43E-18 | positive |
| SMC3 | SPATA5 | 0.708495 | 1.97E-17 | positive |
| SMC3 | TADA2B | 0.70205 | 5.10E-17 | positive |
| SMC3 | TAF3 | 0.78807 | 1.20E-23 | positive |
| SMC3 | TAF5 | 0.801758 | 5.45E-25 | positive |
| SMC3 | TMED8 | 0.706448 | 2.68E-17 | positive |
| SMC3 | UBQLN2 | 0.79817 | 1.25E-24 | positive |
| SMC3 | USP27X | 0.745332 | 5.07E-20 | positive |
| SMC3 | ZBTB2 | 0.757266 | 5.87E-21 | positive |
| SMC3 | ZBTB24 | 0.715872 | 6.45E-18 | positive |
| SMC3 | ZBTB34 | 0.725619 | 1.39E-18 | positive |
| SMC3 | ZBTB39 | 0.749675 | 2.35E-20 | positive |
| SMC3 | ZFP91 | 0.72095 | 2.93E-18 | positive |
| SMC3 | ZNF275 | 0.774422 | 2.10E-22 | positive |
| SMC3 | ZNF281 | 0.730489 | 6.31E-19 | positive |
| SMC3 | ZNF629 | 0.752784 | 1.34E-20 | positive |
| SMC3 | ZNF646 | 0.740688 | 1.14E-19 | positive |
| SMC3 | ZNF740 | 0.722857 | 2.16E-18 | positive |
| SMC3 | ZRANB1 | 0.802143 | 4.98E-25 | positive |
| SMC3 | ZXDA | 0.730323 | 6.48E-19 | positive |
| SMC3 | ZXDB | 0.817124 | 1.25E-26 | positive |
| SOX17 | MYCT1 | 0.757994 | 5.12E-21 | positive |
| SOX2 | C5AR2 | -0.70698 | 2.47E-17 | negative |
| SOX2 | FAM181B | 0.77397 | 2.31E-22 | positive |
| SOX2 | GAL3ST3 | 0.758424 | 4.73E-21 | positive |
| SOX2 | GFER | 0.717922 | 4.70E-18 | positive |
| SOX2 | MAGEF1 | 0.740677 | 1.14E-19 | positive |
| SOX2 | NRARP | 0.75514 | 8.69E-21 | positive |
| SOX2 | PRPF38A | 0.761353 | 2.72E-21 | positive |
| SOX2 | SOX21 | 0.776641 | 1.34E-22 | positive |
| SOX2 | VANGL2 | 0.800174 | 7.89E-25 | positive |
| SOX2 | ZBTB12 | 0.700378 | 6.50E-17 | positive |
| SOX2 | ZNF853 | 0.709486 | 1.70E-17 | positive |
| SOX9 | VANGL2 | 0.714872 | 7.52E-18 | positive |
| SOX9 | ZNF853 | 0.736119 | 2.47E-19 | positive |
| SRF | PATL1 | 0.706691 | 2.58E-17 | positive |
| TCF7L1 | VANGL2 | 0.757964 | 5.15E-21 | positive |
| THAP11 | DDX28 | 0.782067 | 4.34E-23 | positive |
| THAP11 | VPS4A | 0.702025 | 5.12E-17 | positive |
| XRN2 | GID8 | 0.725304 | 1.46E-18 | positive |
| XRN2 | MAPRE1 | 0.750314 | 2.09E-20 | positive |
| XRN2 | MOCS3 | 0.748025 | 3.15E-20 | positive |
| XRN2 | TOP1 | 0.707432 | 2.31E-17 | positive |
| XRN2 | TP53RK | 0.715667 | 6.66E-18 | positive |
| ZBTB33 | CDC5L | 0.775631 | 1.65E-22 | positive |
| ZBTB33 | ETAA1 | 0.750323 | 2.09E-20 | positive |
| ZBTB33 | FAM199X | 0.830707 | 3.28E-28 | positive |
| ZBTB33 | FUBP3 | 0.739833 | 1.32E-19 | positive |
| ZBTB33 | GEMIN5 | 0.724769 | 1.60E-18 | positive |
| ZBTB33 | HNRNPH2 | 0.735453 | 2.77E-19 | positive |
| ZBTB33 | KBTBD7 | 0.704383 | 3.63E-17 | positive |
| ZBTB33 | KIAA1143 | 0.742234 | 8.71E-20 | positive |
| ZBTB33 | KLHL15 | 0.725838 | 1.34E-18 | positive |
| ZBTB33 | LRRC58 | 0.783449 | 3.24E-23 | positive |
| ZBTB33 | MSL2 | 0.713037 | 9.96E-18 | positive |
| ZBTB33 | NUP133 | 0.774616 | 2.02E-22 | positive |
| ZBTB33 | PJA1 | 0.739798 | 1.32E-19 | positive |
| ZBTB33 | PRPF38A | 0.788312 | 1.14E-23 | positive |
| ZBTB33 | PURA | 0.705146 | 3.24E-17 | positive |
| ZBTB33 | RBM27 | 0.759196 | 4.09E-21 | positive |
| ZBTB33 | SASS6 | 0.735936 | 2.55E-19 | positive |
| ZBTB33 | SMARCA5 | 0.798113 | 1.27E-24 | positive |
| ZBTB33 | TADA2B | 0.707758 | 2.20E-17 | positive |
| ZBTB33 | TAF9B | 0.719253 | 3.82E-18 | positive |
| ZBTB33 | VANGL2 | 0.733021 | 4.15E-19 | positive |
| ZBTB33 | ZBTB39 | 0.769202 | 5.97E-22 | positive |
| ZBTB33 | ZFP91 | 0.728166 | 9.22E-19 | positive |
| ZBTB33 | ZNF275 | 0.771335 | 3.91E-22 | positive |
| ZBTB33 | ZNF740 | 0.762487 | 2.20E-21 | positive |
| ZBTB33 | ZNF770 | 0.737852 | 1.85E-19 | positive |
| ZBTB33 | ZXDA | 0.776448 | 1.39E-22 | positive |
| ZBTB33 | ZXDB | 0.820138 | 5.74E-27 | positive |


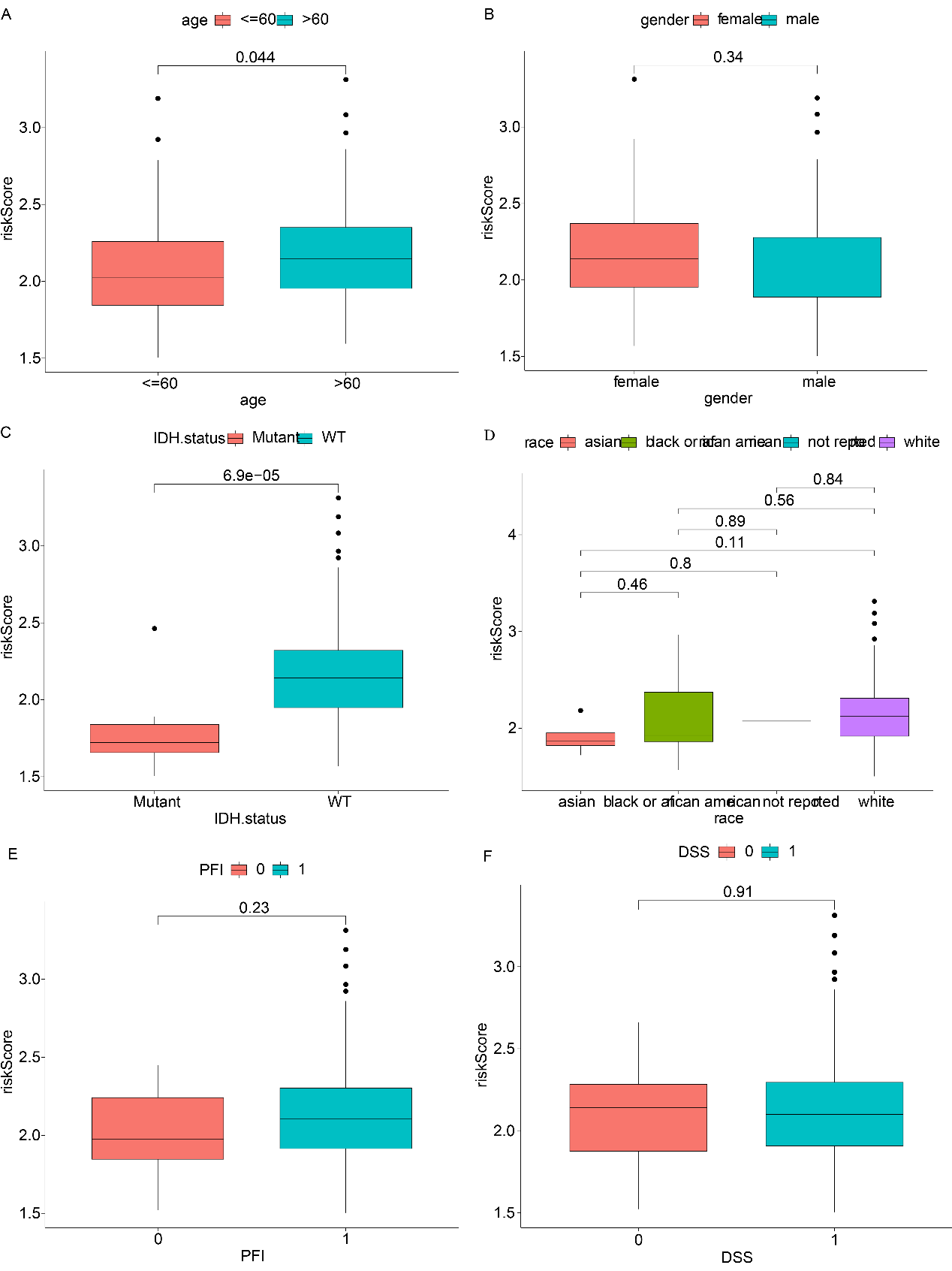


Supplementary Figure 1. The comparison between the risk score and the clinical pathological factors. The comparison of risk scores based on several factors including age (A), gender (B), IDH status (C), race (D), PFI (E), and DSS (F) in the TCGA-glioblastoma dataset. IDH, isocitrate dehydrogenase; PFI, Progression-Free Interval; DSS, Disease-Specific Survival; TCGA, The Cancer Genome Atlas.


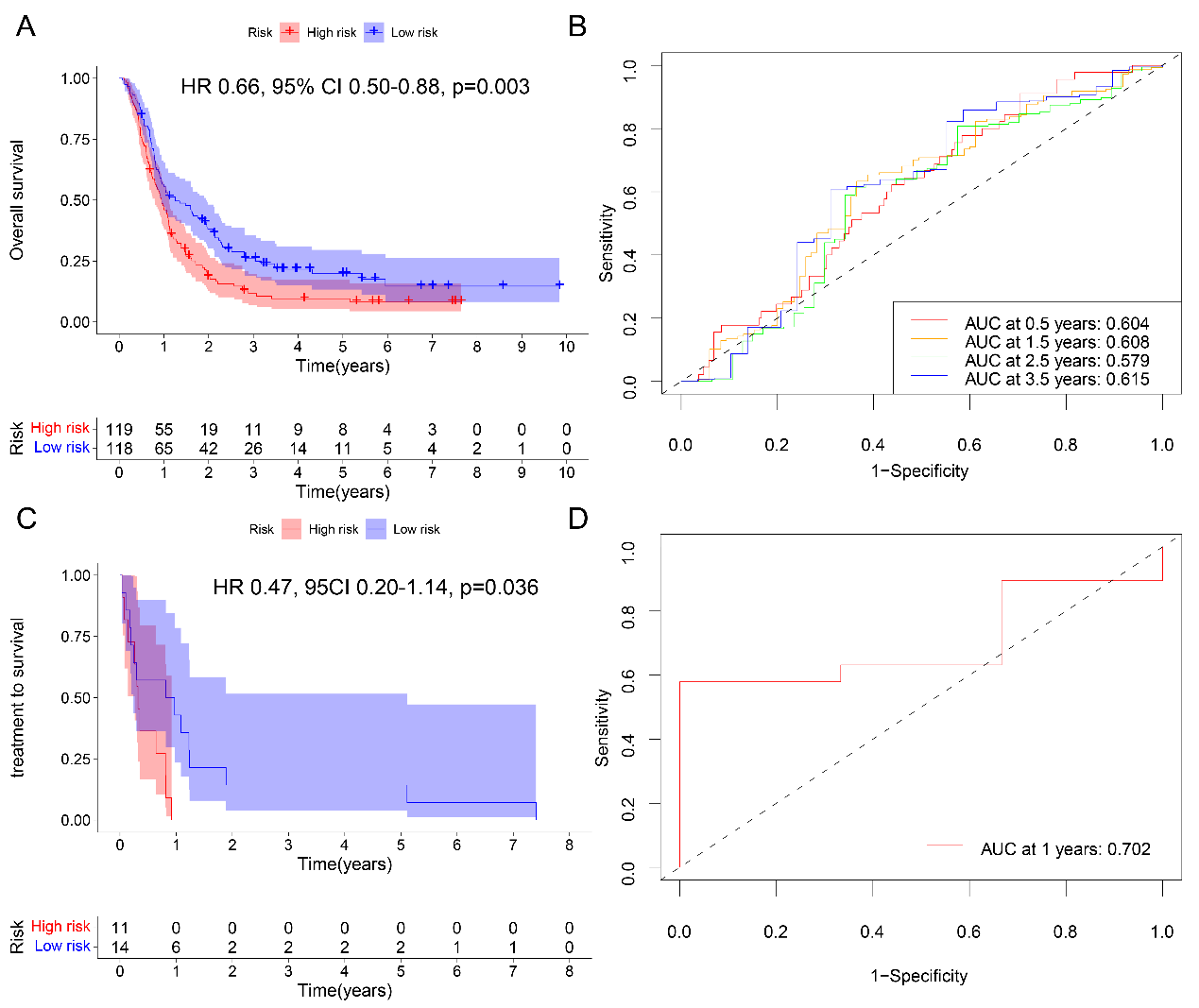


Supplementary Figure 2. Comparison of survival between high and low-risk groups in glioblastoma. Kaplan-Meier survival curves illustrating the overall survival of high- and low-risk groups in both the CGGA-glioblastoma cohort (A) and GSE13041 (GPL570) cohort (C). The ROC curves for years 0.5, 1.5, 2.5, and 3.5 in the CGGA-glioblastoma cohort (B). The ROC curves for 1 year in the GSE13041 (GPL570) cohort (D). ROC, Receiver Operating Characteristic; CGGA, Chinese Glioma Genome Atlas.


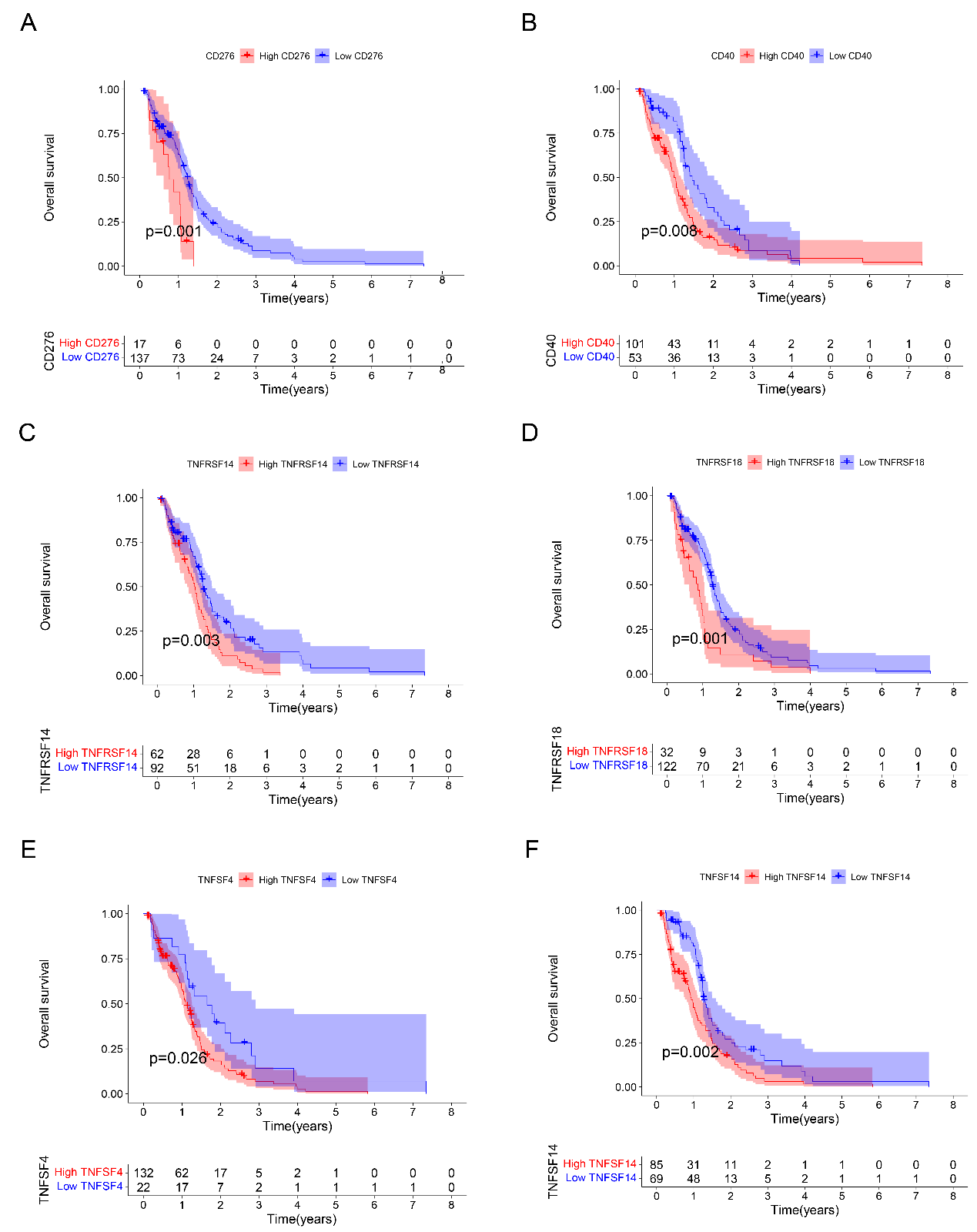


Supplementary Figure 3. Comparison of survival based on high and low model gene expression levels in TCGA-glioblastoma cohort. The Kaplan-Meier survival curves were used to compare the overall survival of patients in the TCGA-glioblastoma cohort based on the expression levels of CD276 (A), CD40 (B), TNFRSF14 (C), TNFRSF18 (D), TNFSF4 (E), and TNFSF14 (F). TCGA, The Cancer Genome Atlas.


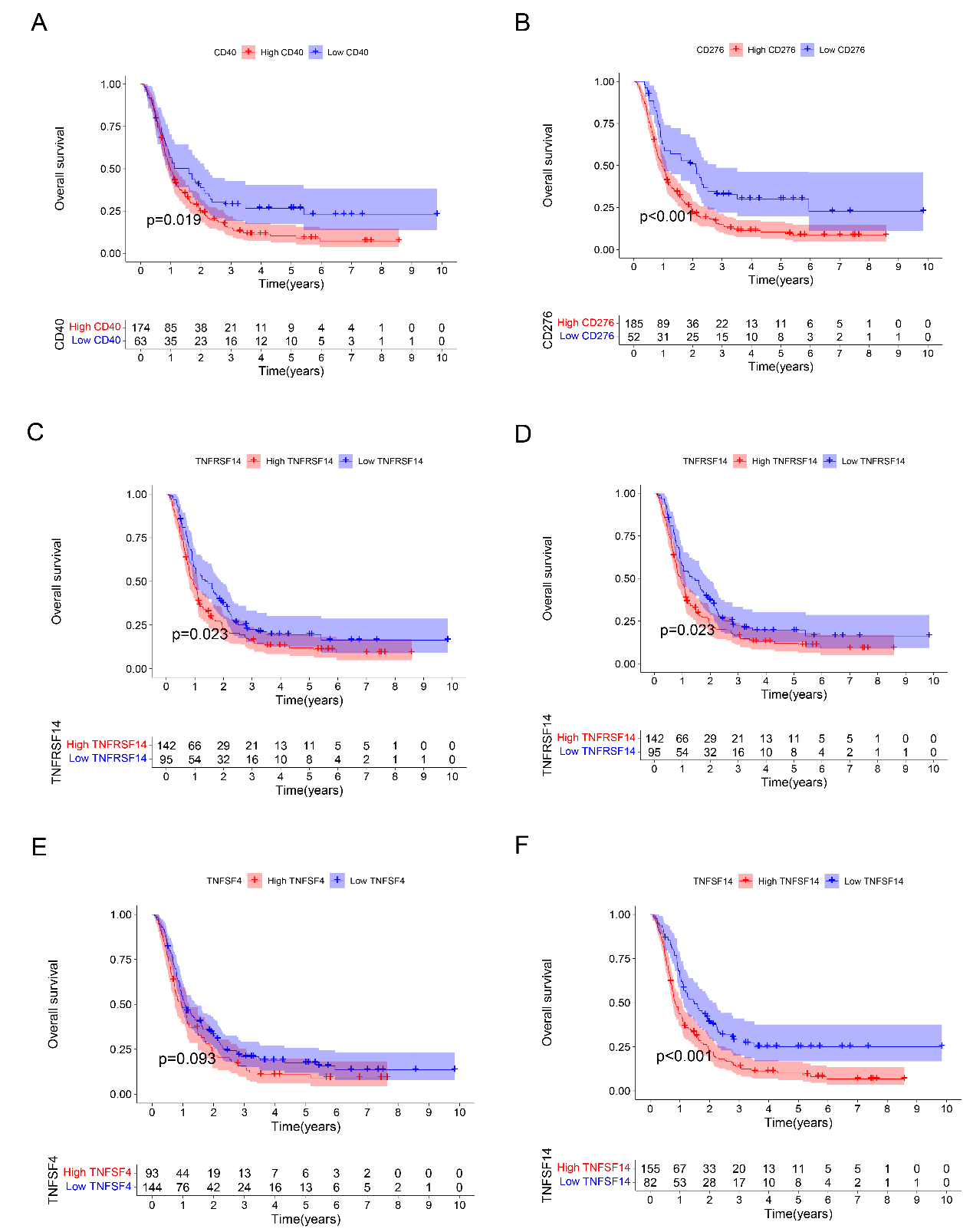


Supplementary Figure 4. Comparison of survival based on high and low model gene expression levels in CGGA -glioblastoma cohort. The Kaplan-Meier survival curves were used to compare the overall survival of patients in the CGGA-glioblastoma cohort based on the expression levels of CD276 (A), CD40 (B), TNFRSF14 (C), TNFRSF18 (D), TNFSF4 (E), and TNFSF14 (F). CGGA, Chinese Glioma Genome Atlas.


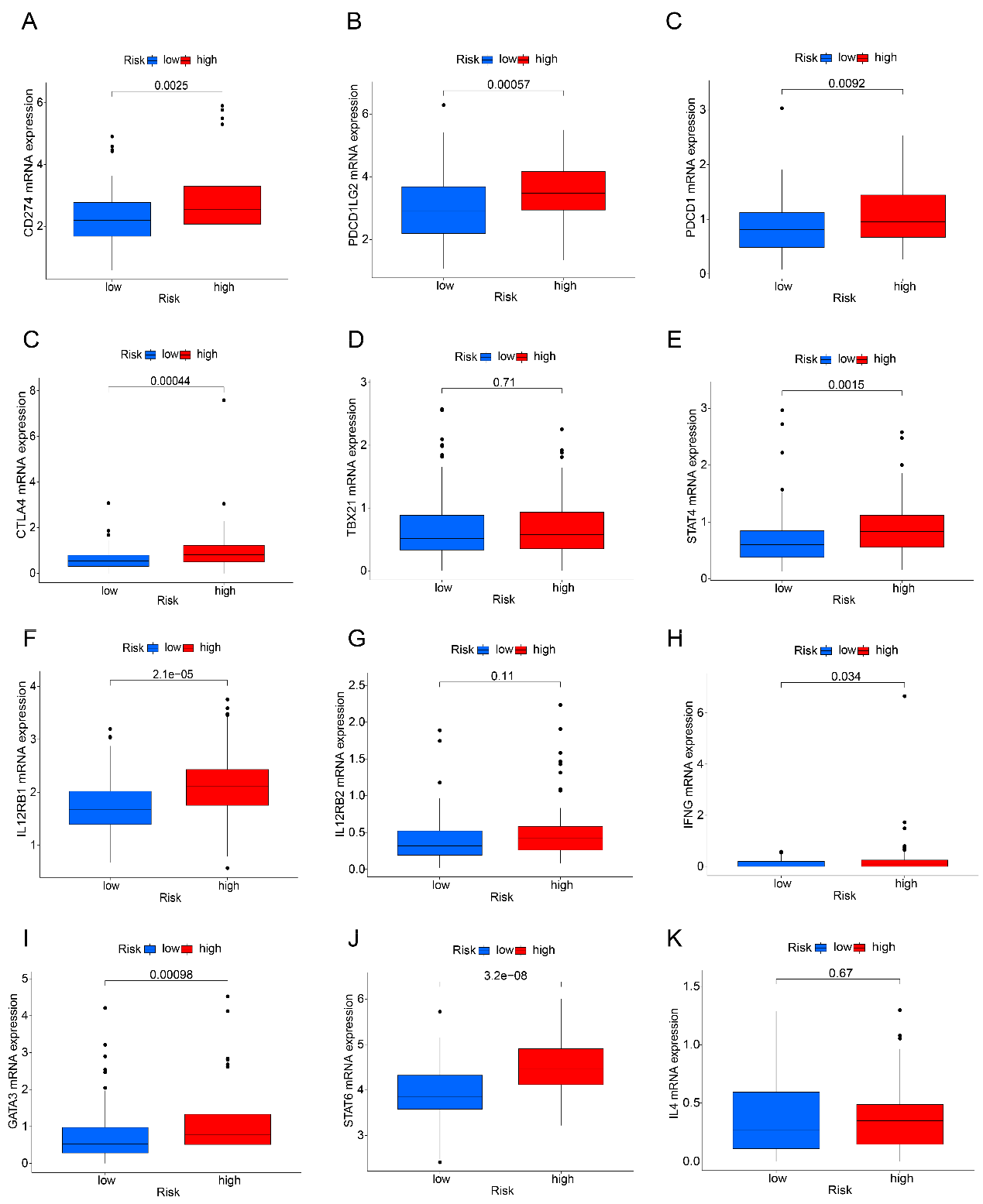


Supplementary Figure 5. Comparison of the expression profiles of Th1 and Th2 marker genes between high and low-risk groups. Comparison of the expression profiles of immune checkpoint genes including PD-L1/CD274 (A), PD-L2/ PDCD1LG2 (B), PD-1/PDCD1 (C), and CTLA4 (D) between high and low-risk groups. Comparison of the expression profiles of Th1 marker genes including TBX21 (D), STAT4 (E), IL12RB1 (F), IL12RB1 (G), and IFNG (H) between high and low-risk groups. Comparison of the expression profiles of Th1 marker genes including TBX21 (A), STAT4 (B), IL12RB1 (C), IL12RB1 (D), and IFNG (H) between high and low-risk groups. Comparison of the expression profiles of Th2 marker genes including GATA3 (I), STAT4 (J), and IL4 (K) between high and low-risk groups.


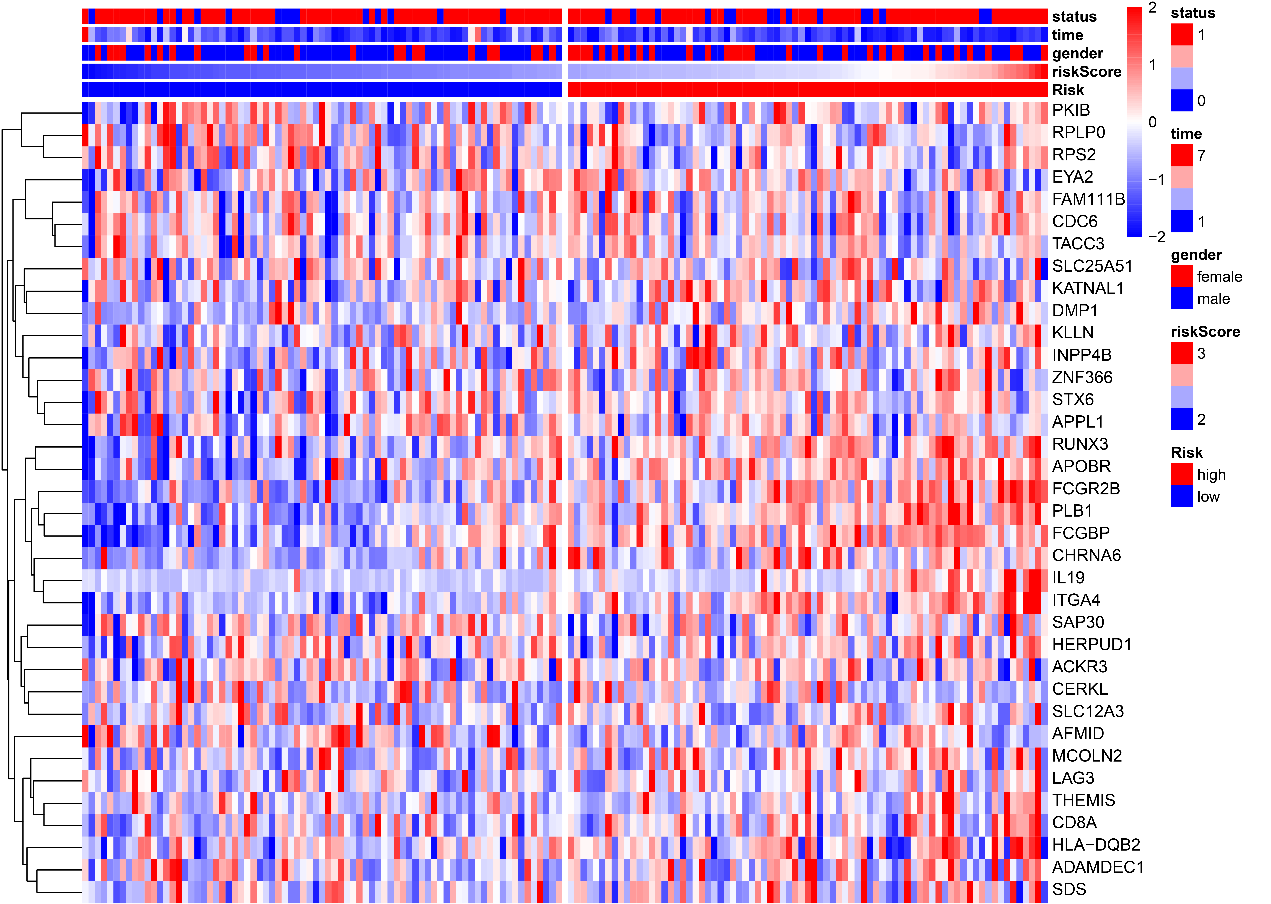


Supplementary Figure 6. The expression of tumour-associated macrophages markers in glioblastoma according to risk, risk score, gender, overall survival and overall survival time.


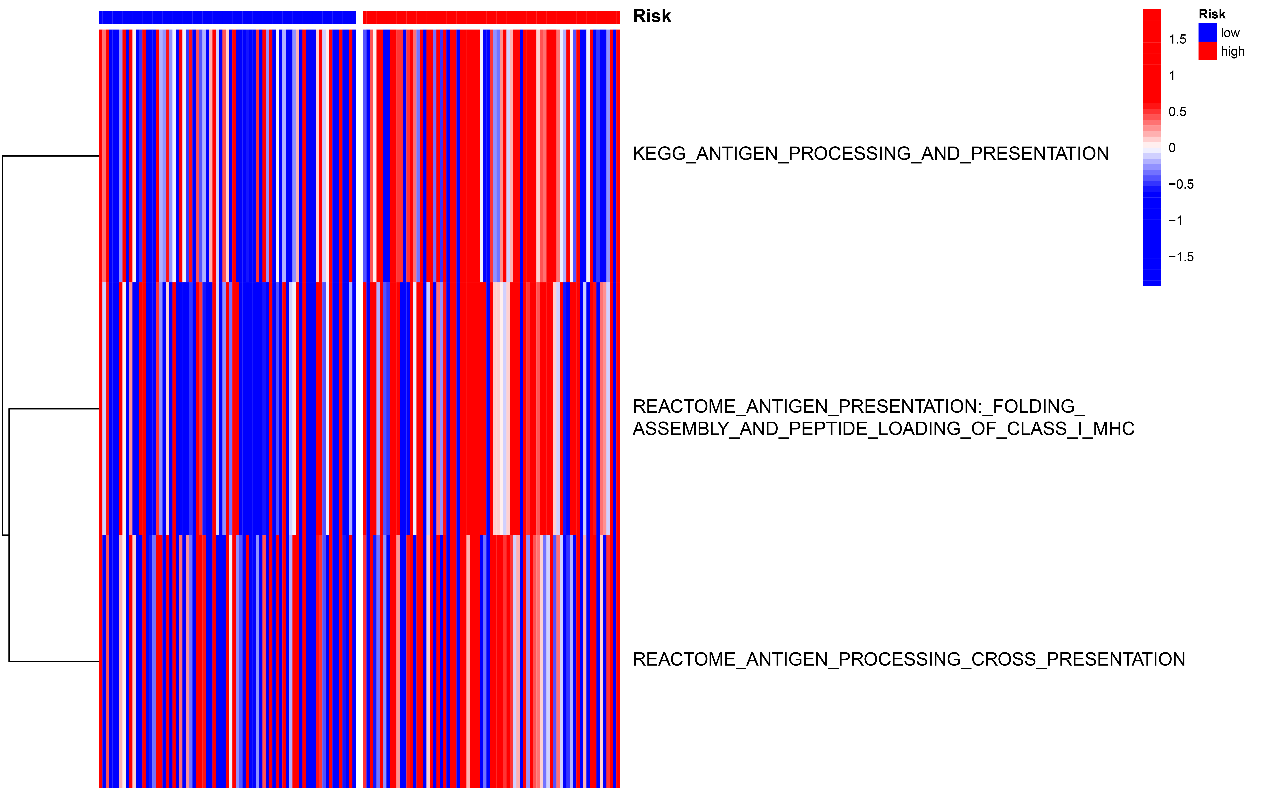


Supplementary Figure 7. Enrichment of antigen presenting process by GSVA based on high and low-risk groups. GSVA, Gene Set Variation Analysis.


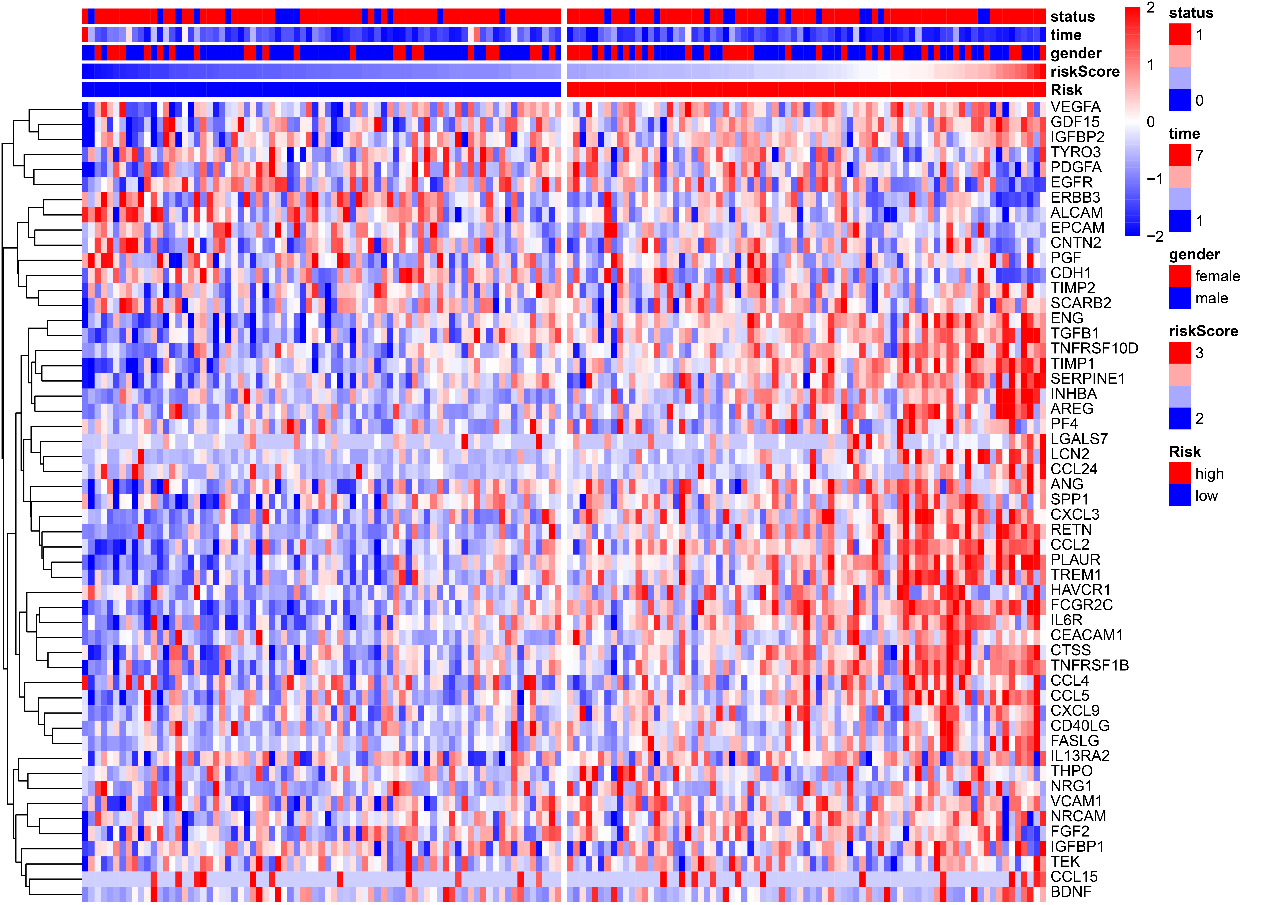


Supplementary Figure 8. The differentially expressed cytokines were analyzed in relation to overall survival status, overall survival time, gender, risk score, and risk.


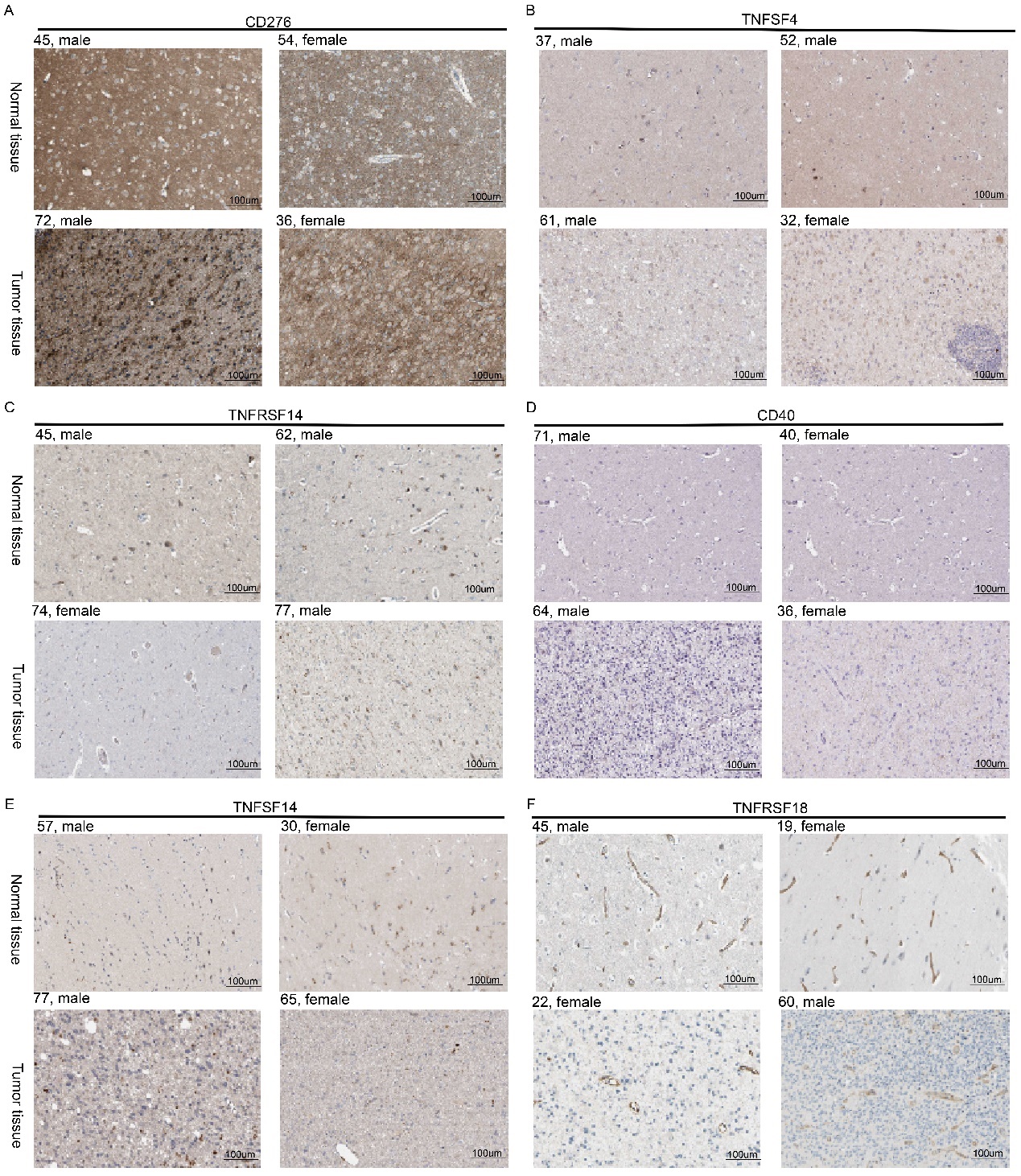


Supplementary Figure 9. Representative IHC staining images of CD276 (A), TNFSF4 (B), TNFRSF14 (C), CD40 (D), TNFSF14 (E), and TNFRSF18 (F) in both normal tissues and glioblastoma samples from the Human Proteome Atlas.
